# GWAS-based polygenic profiling across broad sport categories: shared and category-specific genetic associations with athlete status and polygenic differences from population controls

**DOI:** 10.3389/fspor.2026.1813231

**Published:** 2026-09-30

**Authors:** M. V. Terekhov, G. A. Abushinova, A. A. Mamchur, M. V. Bruttan, I. K. Dzhumaniiazova, M. V. Ivanov, A. V. Zholinsky, A. I. Kadykova, N. S. Gladyshev, R. V. Deev, L. R. Matkava, M. N. Pilipenko, S. I. Mitrofanov, A. A. Ivashechkin, E. D. Maralova, V. S. Yudin, V. V. Makarov, A. A. Keskinov, D. A. Kashtanova, S. M. Yudin, V. I. Skvortsova

**Affiliations:** 1 Federal State Budgetary Institution “Centre for Strategic Planning and Management of Biomedical Health Risks” of the Federal Medical-Biological Agency of Russia (FMBA), Moscow, Russia; 2 National Center of Sports Medicine of the Federal Medical-Biological Agency of Russia (FMBA), Moscow, Russia; 3 Federal Medical-Biological Agency of Russia (FMBA), Moscow, Russia

**Keywords:** GWAS, whole-genome sequencing, polygenic score, sport genetics, athlete status, longevity

## Abstract

Distinct physical demands across sports categories suggest a potential genetic component underlying an athlete's suitability for specific disciplines. To examine this hypothesis, the study analyzed a cohort of 3,676 professional athletes from a range of sports categories, including combat sports (*n* = 875), multi-sports (*n* = 102), speed- and power-based sports (*n* = 146), coordination-based sports (*n* = 630), game-oriented sports (*n* = 1,143), and cyclic sports (*n* = 780). Six genome-wide association studies were conducted to identify genetic variants associated with each sports category, with a general population sample of 21,516 participants as the control group. Comparison of the findings revealed both common and distinct genetic markers across the different sports categories. The findings of each study were used to build polygenic score models to identify the genetic differences between each athlete group and the control. In the internal held-out test set, the models demonstrated varying discriminative abilities, with ROC AUC values of 0.67 for coordination-based sports, 0.75 for combat sports, 0.77 for speed- and power-based sports, 0.82 for multi-sports, 0.68 for game-oriented sports, and 0.67 for cyclic sports. Subsequent validation on a fully independent external sample confirmed the discriminative capacity of the scores for combat sports (0.69), cyclic sports (0.66), coordination-based sports (0.64), and game-oriented sports (0.62), whereas the models for multi-sports (0.53) and speed- and power-based sports (0.38) did not validate externally and were excluded from further analyses. In this study, we present a genetic profiling framework based on exploratory polygenic score modeling that serves as a tool to capture genetic associations with broad sport categories rather than to predict individual athletic success. Additionally, long-term effects of the detected genetic variants were analyzed in a cohort of 2,914 older adults (age ≥ 90 years), in whom polygenic scores for coordination-based, cyclic, and game-oriented sports were higher than in the general population sample. Due to the study's cross-sectional design, these findings are reported as associations with a long-living phenotype, rather than a prediction of lifespan.

## Introduction

Sports play a vital role in the lives of millions of people around the world. Achieving elite athletic performance requires a high level of physical fitness, regular rigorous training, and innate talent. The genetic contribution to elite performance and its utility for sports talent identification remain debated subjects, with a prevailing expert consensus that current genetic testing lacks validated evidence for talent selection or individualized training prescriptions. Understanding how genetics and training shape athletic performance has been a primary focus among sports professionals and scientists alike. Studies have identified over 250 variants across more than 140 genes associated with traits that influence athletic performance. However, only a select number of these variants are crucial in determining the capabilities of elite athletes (1).

Participation in sports typically demands enhanced capabilities, such as endurance, anaerobic capability, muscle recovery, and bone strength. Athletes and non-athletes differ in lung capacity, muscle mass, bone density, hemoglobin levels, and other characteristics (2). These characteristics are also influenced by age-related factors (3, 4).

Each sport presents its own unique set of requirements due to the specific nature of the activities involved. Endurance sports necessitate the ability to sustain a continuous, repetitive pattern of movement over extended periods. In contrast, disciplines focused on speed and power require athletes to generate maximum force in minimal time for explosive movements. Coordination-based sports demand the maintenance of balance and core stability while executing various types of movements. Combat sports require a combination of prolonged static holds, speed, and endurance. Team sports involve adaptability to changing intensities, placing significant demands on the body's sensory systems and requiring exceptional jumping endurance and explosive power. Lastly, multi-sport events integrate the demands of various sports, making them highly physically demanding. As a result, athletes encounter specific physiological constraints based on the demands of their chosen discipline.

Numerous studies have examined the molecular predictors of specific athletic abilities and have identified a number of potentially significant SNPs that may contribute to a predisposition to achieving success in certain sports categories (5, 6). However, there is a paucity of studies that have had a large enough sample of athletes to conduct genome-wide association studies (GWAS), which have great potential as a way of identifying genotypes associated with athletic performance (7).

The objective of this study was to identify genetic variants associated with athlete status across various sports categories by analyzing individual genomic data. Genome-wide association studies were conducted for six sports categories: combat sports, cyclic sports, speed- and power-based sports, coordination-based sports, multi-sports, and game-oriented sports. The findings contributed to the development of polygenic score models that reflect genetic associations with broad sports categories. Additionally, the study explored the relationship between the identified genetic traits and the long-living phenotype (age ≥90 years) and muscle mass preservation in older adults.

## Methods

### Participants

The study included professional athletes from Russian national sports teams who underwent a comprehensive medical examination (CME) at the clinics of the Federal Medical-Biological Agency of Russia (FMBA) in 2021–2022. The CME data were uploaded into the Medical Information and Analytical System of Moscow (MIAS). The following CME data were obtained from the MIAS: body surface area (BSA), body mass index (BMI), weight, height, body girths, right- and left-hand grip strengths, body composition (based on the bioelectrical impedance analysis), and VO_2_ max per 1 kg of body weight. Anthropometric and physical parameters were measured in accordance with the provisions set forth in Decree № 1144n of the Ministry of Health of the Russian Federation dated October 23, 2020. Peripheral venous blood was collected during the CME at the FMBA clinics.

The participants were divided into 6 groups representing their respective sports categories in accordance with the Olympic sports classification used in Russia (8) (Table 1).

**Table 1:** Sports categories represented by the study participants.

Sport category	Disciplines
Combat sports	Belt wrestling
	Judo
	Wrestling
	Taekwondo
	Boxing
	Kurash
	Jujutsu
	Mixed Martial Arts (ММА)
	Karate
Multisports	Triathlon
	Modern pentathlon
	Nordic combined
Speed-and power-based sports	Bobsleigh
	Weightlifting
	Powerlifting
Coordination-based sports	Snowboarding
	Fencing
	Freestyle skiing
	Artistic gymnastics
	Trampolining
	Figure skating
	Synchronized swimming
	Aesthetic group gymnastics
	Skateboarding
	Ballroom dancing
	Ski jumping
Game-oriented sports	Field hockey
	Table tennis
	Badminton
	Curling
	Tennis
	Rugby
	Handball
	Bandy
	Bowling
	Water polo
	Softball
	Volleyball
	Hockey
	Football
	Basketball
Cyclic sports	Diving
	Sledding
	Swimming
	Cyclic
	Canoeing and Kayaking
	Orienteering
	Underwater sports
	Water skiing
	Rowing
	Downhill skiing
	Athletics
	Canoe slalom
	Speed skating
	Biathlon

A population sample of non-athletes was used as the control group. The data for the control groups were obtained from the database of the Centre for Strategic Planning and Management of Biomedical Health Risks of the Federal Medical and Biological Agency of Russia (9). This population sample has been previously described in our previous publication (10). To assess the long-term effects of the detected genetic traits, an analysis was conducted in a sample of older adults aged ≥90 years, which has also been described earlier (11). The sample of long-lived individuals does not overlap with the population cohort serving as controls in the GWAS, yet is ancestrally comparable to it.

### DNA extraction, genome-wide sequencing, and bioinformatic processing

DNA extraction, genome-wide sequencing, and bioinformatic processing were performed at the Federal State Budgetary Institution “Centre for Strategic Planning and Management of Biomedical Health Risks” of the Federal Medical and Biological agency. All blood samples were processed using a standardized protocol. All samples were sequenced on the same platform and processed through a single bioinformatics pipeline under identical parameters, thereby avoiding technical batch effects.

DNA was extracted from whole-blood samples using the QIAamp DNA Mini Kit (Qiagen, Germany). Whole-genome sequencing libraries were prepared using the Nextera DNA Flex Kit (Illumina, USA). The samples were sequenced to 150 bp reads using the NovaSeq 6000 S4 Reagent Kit (300 cycles) (Illumina, USA).

FASTQ files were obtained by demultiplexing the sequencing data in BCL format using bcl2fastq2 Conversion Software v2.20 (Illumina) (12). Sequencing quality was assessed using Sequencing Analysis Viewer v2.4.7 (Illumina) (13). The quality of the FASTQ files was evaluated using FastQC v0.11.9 (14). The reads were aligned to the reference genome, GRCh38.d1.vd1 (15), using the Illumina DRAGEN Bio-IT Platform (v07.021.510.3.5.7) (16). The alignment quality of the BAM files was checked using DRAGEN FastQC v0.11.9 (14), SAMtools v1.13 (17), and mosdepth v0.3.1 (18). All the samples were checked for duplicates, unmapped reads, and other quality metrics. The mean sequencing coverage was at least 30× for all samples.

Small variant calling of up to 50 bp was performed using Strelka2 v2.9.10 (Illumina) (19). CrosscheckFingerprints (Picard) (20), utilizing a haplotype map file, was used to check for duplicates. Ensembl Variant Effect Predictor (VEP v99.2) (21) was used for variant annotation.

A detailed description of participant filtering based on DNA sequencing results and bioinformatic quality control is provided in the Supplementary Methods. All participant counts reported throughout the manuscript are given after applying the described filtering steps.

### Genome-wide association study (GWAS)

A principal component analysis (PCA) was conducted to account for population structure and ethnic diversity. The first 10 principal components were used as covariates in the GWASs.

The following variants were removed:

- Variants violating the Hardy‒Weinberg equilibrium (*p*-value <10^−6^) in the control group;
- Variants with an allele frequency (AF) greater than 0.99;
- Multiallelic variants;
- Variants with a minor allele frequency (MAF) of less than 0.01.

The following logistic regression equation was used to test genome-wide associations:

$$log\left(\frac{\;p}{1-p}\right)=\beta_{0}+\beta_{c}\cdot C+\beta_{g}\cdot G,$$ (1)

where:

$\beta_{0}$ = constant,
$\beta_{c}$ = covariate effect vector,
*C* = covariate vector,
$\beta_{g}$ = genotype effect vector,
*G* = genotype vector.

Genotypes were encoded additively as 0, 1, and 2 for the number of alternative alleles.

All calculations were performed using Statsmodels v0.12.2 (Python) and Spark Cluster parallelization. Age, sex, and the first 10 principal components were used as covariates. Variants with *p*-values of less than 5.0 × 10^−8^ were considered to be significant; variants with *p*-values of less than 5.0 × 10^−5^ were considered to be sub-significant. The results were visualized using the LocusZoom Javascript library.

### Comparison of GWAS findings

To compare the GWAS findings, variants with a *p*-value of less than 10^−5^ in at least one of the studies were selected. When analyzing all six GWAS simultaneously, the results were visualized in a UMAP space, allowing for the identification of variant clusters. Each cluster was annotated with the names of genes most frequently represented within each cluster.

### Polygenic score models

Polygenic score models were built using the respective GWAS findings for each of the six sports categories to quantify genetic differences between each athlete group and the control. To address overrepresentation, participants were randomly removed from the control group, ensuring a 1:2 case-to-control ratio. Then, the sample was divided into training and internal test sets in a 4:1 ratio.

The regression models were built in the Python v3.8 programming language using the scikit-learn library (the LogisticRegression class). The regression coefficients were calculated by minimizing the sum of the squared difference between the observed and predicted values and adding the squared sum of values (ridge regression) and weights (lasso regression) as a combined “penalty” for regularization:

$$loss=\Sigma_{i}\;(y_{i}-\hat{y_{i}}{)}^{2}+0.5(1-\alpha )\lambda \Sigma_{j}\;\beta_{j}^{2}+\alpha \lambda \Sigma_{j}\;\beta_{j}\vee ,$$ (2)

where loss is the loss function to be minimized during the training, y is the observed values, $\hat{y_{i}}$ is the predicted values, β is the model coefficients, *λ* is the regularization parameter, and *α* is the lasso regularization weight. All model parameters remained unstandardized.

Variants for the model were selected based on their *p*-value (<10^−5^) in the respective GWAS. Sex was used as a covariate, along with the first 10 principal components.

For the final hyperparameter tuning, we used random search of an n-number of parameters based on their significance in the final model that was calculated via Fisher's exact test (the first *n* when sorted in descending order), the regularization parameter across a uniform *λ* grid of 50 values from the 10^−5^:10^3^ range, ridge regularization or a combination of ridge and “lasso” regularizations, and, if necessary, “lasso” regularization weights used in the loss function (a uniform grid with *α* ranging from 0.1 to 1). The optimal parameter value was selected by maximizing the area under the curve (AUC). Each model was subjected to a 10-fold cross-validation for the target variable on a validation set of 10% of the training dataset. Table 2 presents the hyperparameter values for each model.

**Table 2:** Hyperparameters and performance metrics of polygenic score models.

Sports category	Number of variables	*λ*	Regularization function	*α*	F1*		ROC AUC		Precision*		Recall*	
					Test	Train	Test	Train	Test	Train	Test	Train
Coordination-based sports	38	0.0066	ridge	0	0.50	0.58	0.67	0.74	0.45	0.53	0.56	0.64
Combat sports	54	0.0021	ridge and lasso	0.4	0.60	0.62	0.75	0.78	0.55	0.57	0.66	0.68
Speed- and power-based sports	45	0.0139	ridge and lasso	0.5	0.61	0.83	0.77	0.94	0.61	0.78	0.61	0.89
Multi-sports	97	2.6827	ridge	0	0.55	0.87	0.82	0.96	0.57	0.88	0.53	0.86
Game-oriented sports	34	54.2868	ridge	0	0.58	0.54	0.68	0.69	0.49	0.44	0.72	0.68
Cyclic sports	27	0.0045	ridge	0	0.53	0.57	0.67	0.73	0.46	0.49	0.64	0.67

* F1-score, precision and recall were calculated at the 0.5 probability threshold. Test = internal hold-out set.

The final probability of an athlete representing a given sports category was calculated via the LogisticRegression built-in predict_proba() method using the formula:

$$P=\frac{1}{1+e^{-(\Sigma_{i}\beta_{i}*g_{i}+\beta_{0})}},$$ (3)

where $g_{i}$ is the predictor value, $\beta_{i}$ is the predictor coefficient, and $\beta_{0}$ is the intercept.

To construct the radar chart, calculated probability values (*P*) obtained across all six developed models were used for each athlete. The chart was generated via the plotly library.

### Statistical analysis

The data were analyzed in Python 3.9.12. Anthropometric data across the sports categories were compared using regression analysis, with the category as the target variable and the examined parameters, sex, age, and BMI, as predictors. For pairwise comparisons, one sports category was coded as 1 and the other was coded as 0. Figure 1 shows the results of the pairwise comparisons. For one-vs.-all comparisons, the compared category was coded as 1, and all other categories were coded as 0. Supplementary Table S1 provides the results of the one-vs.-all comparisons.

**Figure 1:**
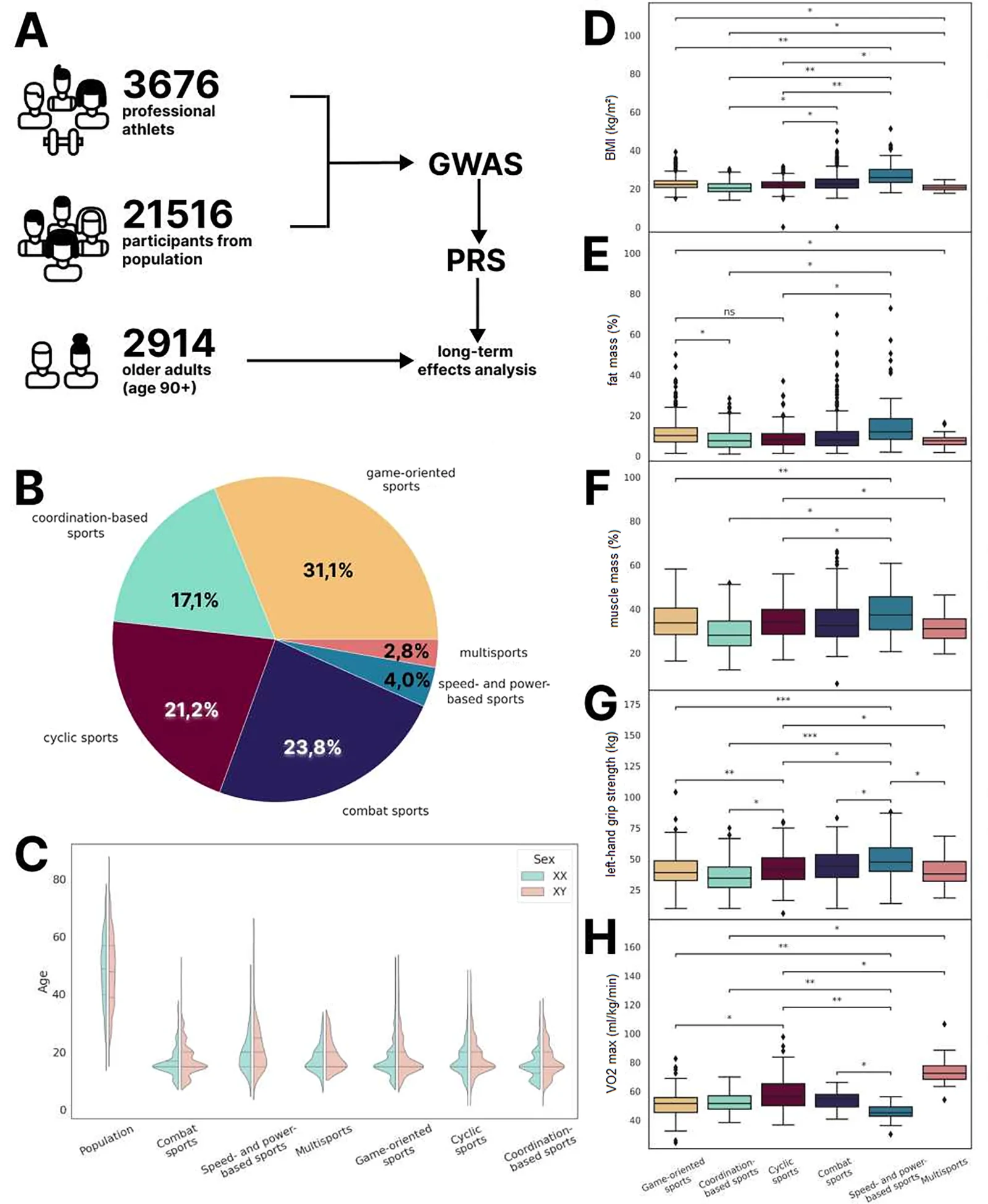
Case group characteristics. **(A)** Main types of available data. **(B)** Representation of various sports categories in the case group. **(C)** Sex and age for the case and control groups. **(D–H)** Anthropometric and physical characteristics of athletes from various sports categories: BMI, fat mass, muscle mass, left-hand grip strength, and VO_2_max.

Regression analysis was also carried out to search for associations between polygenic scores and anthropometric and physical parameters. The target variable was the examined parameter, and the predictors were the polygenic scores generated by each model, age, and BMI.

All *p*-values were subjected to the Bonferroni test for multiple comparisons.

The seaborn and plotly libraries were used for data visualization.

## Results

### Participants

The study included a case group of 3,676 athletes from Russian national sports teams [men = 1,955 (53%); women = 1,721 (47%)], representing combat sports (*n* = 875), multi-sports (*n* = 102), speed- and power-based sports (*n* = 146), coordination-based sports (*n* = 630), game-oriented sports (*n* = 1,143), and cyclic sports (*n* = 780) (Figure 1B). A population sample of 21,516 participants served as the control group (men = 9,236 (43%); women = 12,280 (57%) (Figure 1C). Figure 1 and Supplementary Table S1 present the main anthropometric and physical characteristics of the athletes.

### Genome-wide association study for six sports categories

Six GWASs were conducted to identify genetic variants associated with each sports category, with athletes from the corresponding category serving as the case group and the population sample serving as the control group. It is important to note that national sports teams predominantly consist of younger individuals, which does not accurately represent the average age distribution in the general population. Consequently, age was not included as a covariate in the analysis. Adjustments were limited to sex and the first 10 principal components that capture the genetic structure of the population. The GWAS findings are presented as Manhattan plots in Supplementary Figures S1–S6, QQ-plots are presented in Supplementary Figures S7–S12. Table 3 provides the list of variants that passed the genome-wide significance threshold.

**Table 3:** Significant findings in each of the six GWASs.

Chromosome	Position	Ref. allele	Alt. allele	Allele frequency, control	Allele frequency, case	Regression *p*-value	Odds ratio	Gene	DBSNP ID
Multisports									
4	179677720	A	G	0.011	0.059	1.10*10^−8^	5.82		rs138125544
4	179699161	A	G	0.011	0.064	6.08*10^−10^	6.32		rs151237543
4	179711631	T	C	0.011	0.064	6.21*10^−10^	6.32		rs180831871
10	25714274	A	T	0.009	0.054	6.99*10^−9^	6.33	LINC00836	rs144380919
3	44009227	C	T	0.014	0.064	1.09*10^−8^	5.25		rs148699337
3	117435496	C	T	0.01	0.059	1.81*10^−9^	6.44		rs77475812
3	117447614	C	T	0.009	0.059	1.62*10^−9^	6.47		rs79605040
3	117460503	A	G	0.01	0.059	1.90*10^−9^	6.42		rs115472693
3	117463165	A	C	0.015	0.069	2.79*10^−8^	4.67		rs114773681
3	117488016	C	T	0.01	0.056	2.12*10^−9^	6.39	LOC105374056	rs77411964
16	24771571	G	GTT	0.008	0.049	4.87*10^−8^	6.08	TNRC6A	rs879783845
18	3438596	A	C	0.048	0.147	8.31*10^−9^	3.30	TGIF1	rs7226405
Combat sports									
5	125304962	G	T	0.008	0.017	4.90*10^−8^	2.95	LINC02240	rs149865510
1	117681976	T	C	0.074	0.115	4.18*10^−8^	1.54		rs1539441
10	5052195	G	A	0.146	0.218	1.64*10^−8^	1.42	AKR1C3	rs11599072
11	85829059	T	G	0.007	0.023	2.05*10^−8^	2.70	SYTL2	rs12577360
Coordination-based sports									
4	169041979	C	T	0.008	0.025	4.84*10^−9^	3.03		rs144318129
8	87135486	A	G	0.01	0.031	7.79*10^−10^	2.85	CNBD1	rs117223843
8	87225612	C	G	0.01	0.03	9.64*10^−9^	2.69	CNBD1	rs192088146
8	87228499	G	C	0.009	0.027	4.13*10^−8^	2.68	CNBD1	rs534226114
18	7086629	A	C	0.015	0.037	9.15*10^−10^	2.68	LAMA1	rs2058218638
20	30934072	C	A	0.012	0.033	2.13*10^−10^	2.91		rs1385732528
Cyclic sports									
5	152479947	C	G	0.011	0.030	1.79*10^−8^	2.51		rs75413073
2	164238446	G	C	0.007	0.021	4.31*10^−8^	2.78		rs562552210
22	48679098	T	C	0.126	0.094	2.13*10^−8^	0.57	TAFA5	rs775815705
Speed- and power-based sports									
11	90518893	C	G	0.013	0.055	2.07*10^−9^	5.06	DISC1FP1	rs867704466
7	107944887	C	A	0.009	0.045	4.28*10^−8^	5.11	LAMB1	rs144534951
11	107700896	C	CA	0.022	0.075	6.09*10^−9^	3.69		
6	39284373	G	A	0.009	0.045	9.79*10^−9^	5.23		rs149704888
12	67879719	T	G	0.014	0.058	6.34*10^−9^	4.56		rs1905446
2	80374683	G	A	0.008	0.045	1.81*10^−8^	4.96	CTNNA2	rs189583925
2	80419158	G	T	0.011	0.051	4.79*10^−8^	4.32	CTNNA2	rs185777639
Game-oriented sports									
6	170079158	C	CG	0.125	0.085	1.01*10^−9^	0.63		rs71010624
8	55075004	C	CT	0.009	0.022	2.61*10^−9^	2.45	LOC107986887	rs1180116457
20	30932028	A	T	0.01	0.024	4.59*10^−11^	2.63		rs1029104728
20	30933164	A	G	0.014	0.03	7.69*10^−9^	2.19		rs1039083409
20	30934072	C	A	0.012	0.028	1.09*10^−11^	2.53		rs1385732528
18	7086629	A	C	0.015	0.037	1.98*10^−14^	2.6	LAMA1	rs2058218638

The frequency of occurrence of significant variants differed substantially between the case and control groups. Moreover, many variants are extremely rare, both in the population sample (≈1% frequency of occurrence) and in the gnomAD database for Europeans (Supplementary Table S2). However, carriers of these alleles were more common among athletes. Expectedly, the odds ratio of such variants was more than 1, i.e., they were associated with a particular sports class. However, two significant variants were associated with the control group, namely rs775815705 in the *TAFA5* gene and rs71010624 in the intergenic region on chromosome 6.

Importantly, only two variants—rs2058218638 in the *LAMA1* gene and rs1385732528 in an intergenic region—were significant in two GWASs: in the GWAS for coordination-based sports and the GWAS for game-oriented sports. To explore the genetic overlap among the six athlete categories, we performed a UMAP projection based on the sub-significant GWAS signals (*p* < 10^−5^) from each scan. Figure 2 presents the resulting UMAP visualization, which revealed distinct genetic predispositions among athletes from different sports categories. Most variants included in the analysis passed the threshold of a *p*-value of <10^−5^ in only one GWAS. However, a small cluster of variants, referred to as a shared cluster, was detected across all GWASs (Figure 2). It contained SNPs in the *CPLX1, TAFA5, JPH3*, *LAMA1,* and other genes. These variants passed the genome-wide significance threshold in one or two GWASs and passed the sub-significance thresholding in others, indicating their potential to be significant in a larger sample size. For example, the above-mentioned rs2058218638 in the *LAMA1* gene was in the shared cluster and demonstrated the sub-significant *p*-value in the GWASs for cyclic sports (*p*-value = 1.1*10^−6^) and combat sports (*p*-value = 6.4*10^−6^). Therefore, our findings indicate that different sports categories display both common and unique genetic predictors.

**Figure 2:**
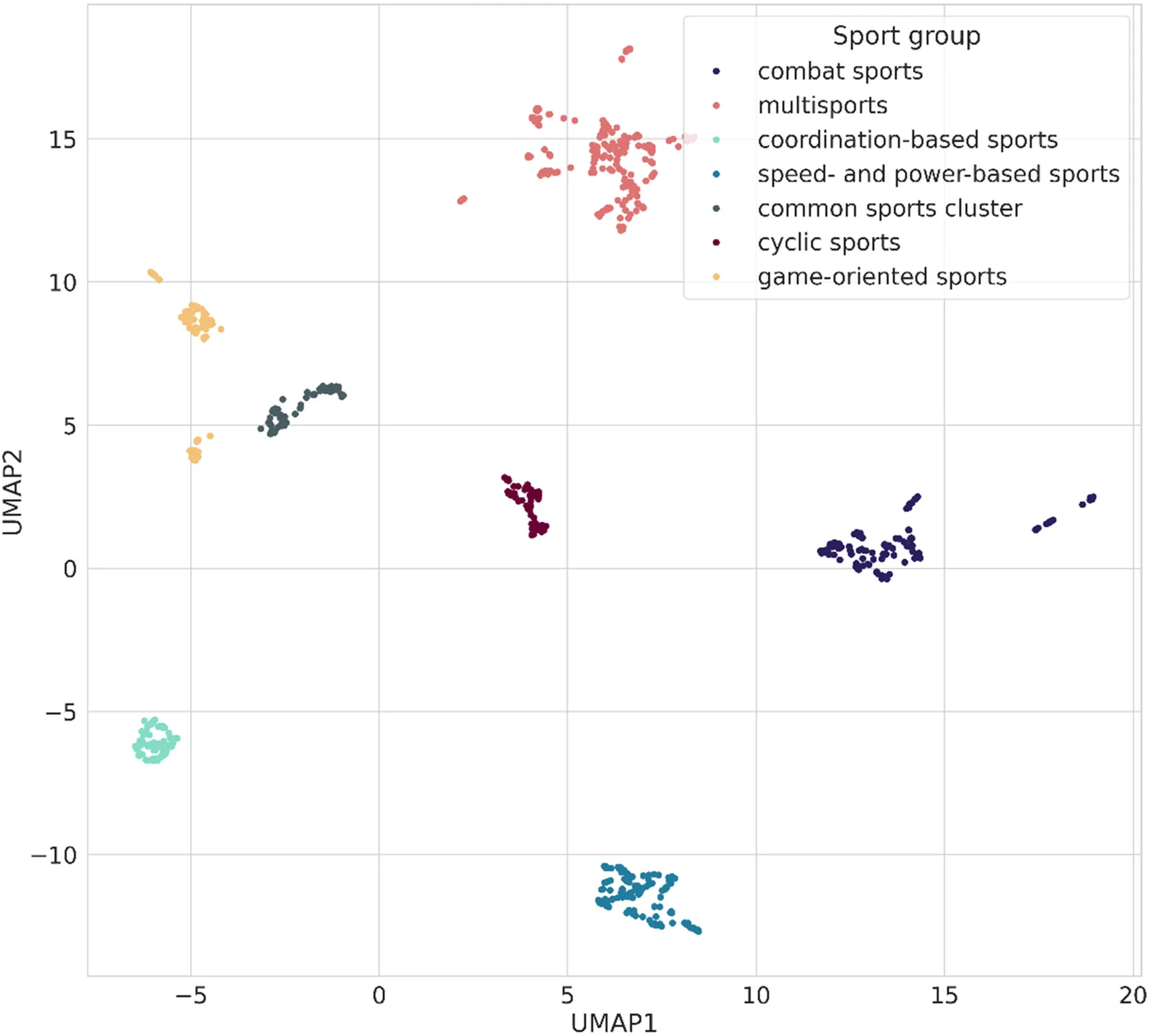
UMAP visualization of genetic overlap among six athlete categories.

### Polygenic score models

Polygenic score models were built using the respective GWAS findings to assess genetic differences between each athlete group and the control. Supplementary Figure S13 shows the ROC curves for the models' training and testing. The average AUC in the in-sample test set was 0.76, with 0.67 for coordination-based sports, 0.75 for combat sports, 0.77 for speed- and power-based sports, 0.82 for multi-sports, 0.68 for game-oriented sports, and 0.67 for cyclic sports (Figure 3A). Additional performance metrics (F1-score, precision, and recall) are presented in Table 2.

**Figure 3:**
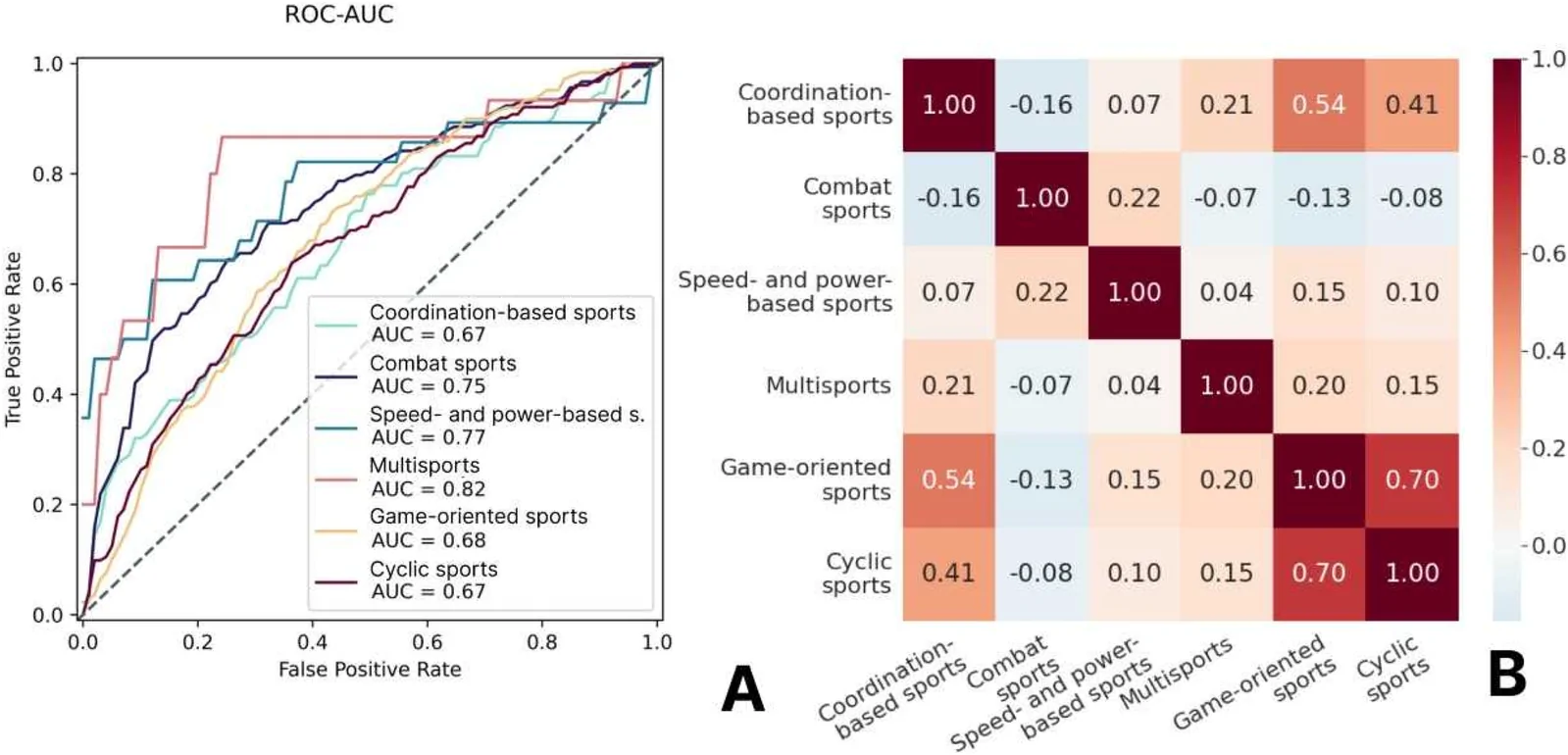
Polygenic scores. **(A)** Results of polygenic score model testing. **(B)** Correlation matrix of polygenic scores of the entire sample (case and control).

Supplementary Tables S3–S8 present the parameters that were predictors in each model. Only parameters optimizing the models' performance were selected, and parameters with zero regression coefficients were excluded. The obtained polygenic score models were applied to the entire study sample, i.e., the case and control groups, and a correlation matrix of the obtained scores was built (Figure 3B). Polygenic scores for game-oriented sports, cyclic sports, and coordination-based sports were strongly correlated, and the corresponding models included a fairly large number of SNPs from the shared cluster shown in Figure 2. However, they also included SNPs that were specific to their sports categories, facilitating their differentiation. These three models also showed a weak positive correlation with speed- and power-based sports and multi-sports. Scores for combat sports, on the contrary, exhibited a weak negative correlation with all other scores, except for speed- and power-based sports.

To validate the constructed models and assess their predictive ability without the risk of data leakage, we used sequencing results from a sample that was not previously included in the GWAS. The participants of this sample represent combat sports (*n* = 100), multi-sports (*n* = 59), speed- and power-based sports (*n* = 100), coordination-based sports (*n* = 100), game-oriented sports (*n* = 100), and cyclic sports (*n* = 100); additionally, 500 individuals constitute a population control sample. The developed models have the following ROC AUC values in this sample: combat sports 0.69, multi-sports 0.53, speed- and power-based sports 0.38, coordination-based sports 0.64, game-oriented sports 0.62, and cyclic sports 0.66.

Based on exploratory polygenic modeling and comparative genetic analyses between each athlete group and the control, we present a polygenic profiling framework that captures genetic variants associated with specific sport disciplines. These profiles are descriptive tools and are not intended to assess an individual's aptitude for specific sports or their athletic success. Figure 4 shows profiles of nine individuals, six of whom are professional athletes from various sports categories (Figures 4A–F), while three are participants from the control group (Figures 4G–I).

**Figure 4:**
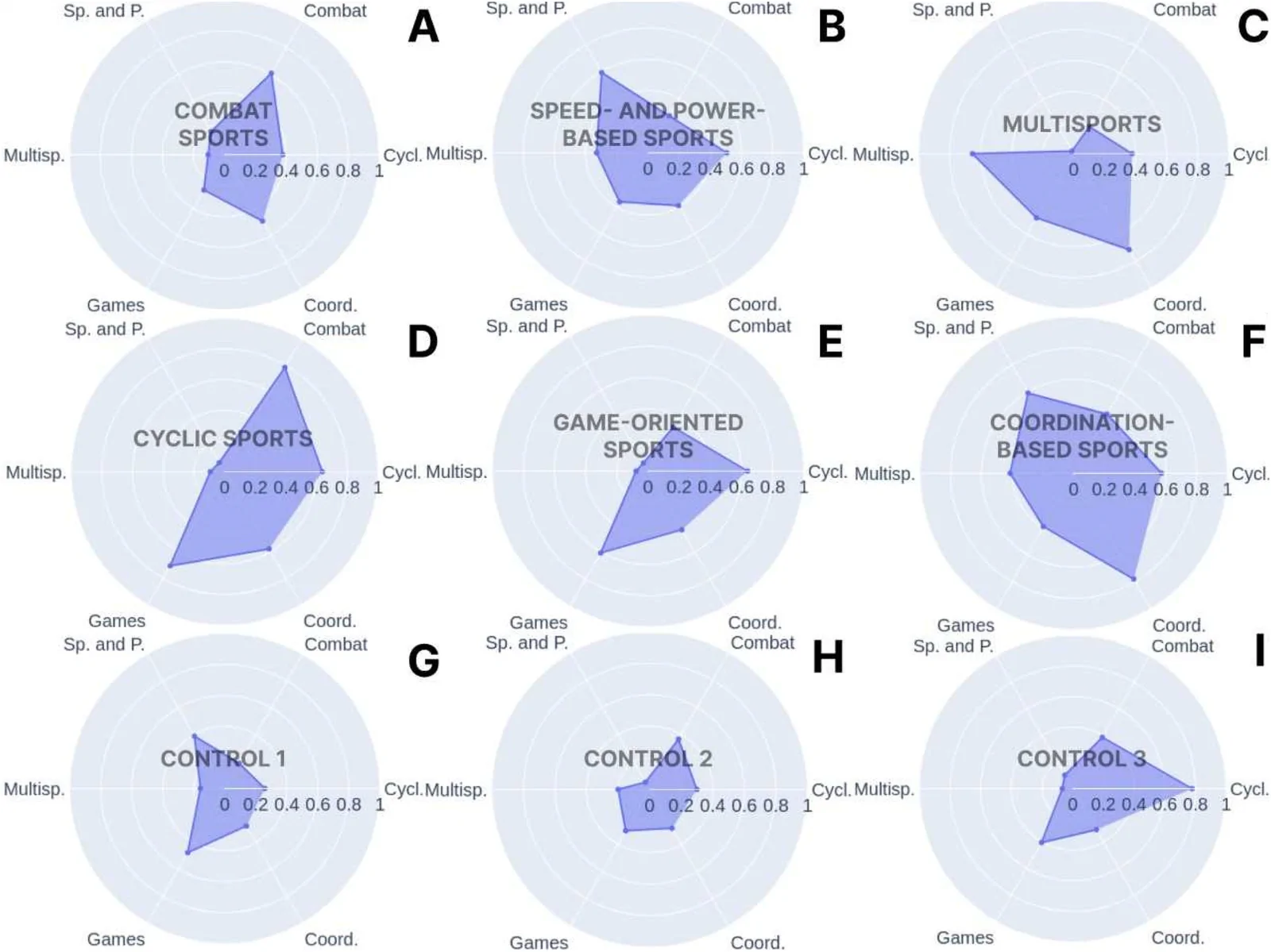
Polygenic profiles showing genetic similarities between each broad sports category and the control. **(A)** Professional athlete from the combat sports category. **(B)** Professional athlete from the speed- and power-based sports category. **(C)** Professional athlete from the multisports category. **(D)** Professional athlete from the cyclic sports category. **(E)** Professional athlete from the game-oriented sports category. **(F)** Professional athlete from the coordination-based sports category. **(G–I)** Participants from the control group.

Professional athletes scored high not only for their respective sports category but also for other categories (Figures 4C–F), which is consistent with the above correlation matrix. Participant Control 3 from the control group (Figure 4I) scored higher for cyclic sports.

Based on this observation and the general understanding of a genetic predisposition to high-performance sports, a hypothesis was put forward that polygenic scores may be reflective of certain physical characteristics. To test this hypothesis, a regression analysis was conducted to examine the association between the polygenic scores and some anthropometric characteristics. Table 4 presents the findings of this analysis.

**Table 4:** Association between polygenic score and athletes' physical characteristics.

Characteristic	Coordination-based sports		Combat sports		Speed—and power-based sports		Multi-sports		Game-oriented sports		Cyclic sports	
	Coefficient	*p*-value*	Coefficient	*p*-value*	Coefficient	*p*-value*	Coefficient	*p*-value*	Coefficient	*p*-value*	Coefficient	*p*-value*
BSA	0.02	1	0.19	2.5*10^−20^	0.04	1	0.03	1	0.29	9.2*10^−26^	0.30	7.7*10^−56^
Height. standing	2.01	1	11.6	1.7*10^−21^	2.35	1	1.78	1	18.7	5.8*10^−32^	19.52	6.8*10^−68^
Chest girth during breathing pause	−5.81	1	10.7	5.8*10^−5^	0.89	1	−2.11	1	1.28	1	4.36	1
Chest girth during inhalation	−2.64	1	8.98	9.7*10^−20^	0.89	1	−0.82	1	5.57	0.001	7.70	3.3*10^−15^
Chest girth during exhalation	−8.18	1	6.88	1	−0.55	1	−3.36	1	−0.26	1	5.15	1
Right forearm girth. flexed	−1.7	0.002	4.72	2.7*10^−53^	0.51	1	−0.62	1	1.95	0.0003	3.09	1.8*10^−23^
Right forearm girth. relaxed	−1.11	0.21	3.23	1.7*10^−30^	0.17	1	−0.64	1	1.82	8.9*10^−5^	2.37	4.7*10^−17^
Right shin girth	1.19	0.424	−0.48	1	0.17	1	0.16	1	3.06	3.0*10^−11^	2.64	4.3*10^−16^
Hip girth	5.58	1.7*10^−8^	−5.26	3.1*10^−12^	−1.04	1	0.36	1	7.79	2.0*10^−15^	4.99	9.6*10^−12^
Neck girth	−3.97	1.4*10^−14^	8.1	7.08*10^−107^	1.04	0.411	−1.62	0.059	−0.07	1	2.59	3.0*10^−10^
Waist girth	−2.38	0.159	7.16	9.2*10^−34^	0.65	1	−0.41	1	4.84	4.0*10^−8^	6.24	3.9*10^−27^
Left-hand grip	−10.13	7.8*10^−9^	23.25	3.4*10^−83^	4.58	0.006	−1.21	1	4.71	0.589	12.22	5.7*10^−23^
Right-hand grip	−10.1	2.2*10^−8^	22.4	2.1*10^−73^	3.95	0.085	−1.67	1	5.98	0.049	13.0	2.9*10^−25^
Hip-to-waist ratio	0.02	1	−0.049	0.135	−0.02	1	0.006	1	0.001	1	−0.02	1
Fat percentage	8.71	6.8*10^−18^	−16.98	4.1*10^−118^	−2.43	0.064	2.68	0.5	0.43	1	−5.83	3.2*10^−13^
Visceral fat percentage	19.51	3.5*10^−6^	−26.45	6.1*10^−19^	−2.37	1	7.19	1	10.93	0.485	−3.00	1
Muscle mass	−2.67	0.61	12.45	5.3*10^−64^	1.93	0.621	−0.93	1	7.95	8.2*10^−14^	11.19	1.1*10^−54^
Fatty mass	5.74	2.4*10^−15^	−10.5	3.5*10^−87^	−1.49	0.341	1.45	1	2.01	0.788	−2.09	0.01
Respiratory function/Vital lung capacity	−0.57	1	1.72	1	−0.2	1	1.38	1	3.03	1	2.63	1
Vo_2_max per 1 kg of body weight	−2.78	1	13.12	0.015	−2.1	1	5.54	1	−1.28	1	5.93	1

* Adjusted for age, BMI, and multiple testing.

Polygenic scores associated with game-oriented sports and cyclic sports were linked to larger girth measurements, increased body surface area, greater grip strength, and higher muscle mass. In contrast, polygenic scores for coordination-based sports demonstrated correlations with smaller shoulder and neck girth measurements, larger thigh girth measurements, lower grip strength, and higher adipose tissue compared to other athletic categories. Polygenic scores for combat sports were correlated with increased height, larger shoulder and neck girth measurements, smaller hip girth measurements, enhanced grip strength, and increased lean muscle mass, alongside a reduction in adipose tissue mass. Polygenic scores for multi-sports and speed- and power-based sports did not show significant associations with the examined anthropometric and physical characteristics, with the exception of grip strength, which exhibited a notable positive correlation with scores for speed- and power-based sports.

The results obtained are highly consistent with the anthropometric description of sports categories presented in Supplementary Table S1 and align with the established evidence for polygenic profiling's ability to capture phenotypic differences across broad sports categories at the group level. However, the scores for multi-sports and speed- and power-based sports do not reflect any specific characteristics, possibly due to both the general phenotypic heterogeneity characteristic of athletes from these categories and the insufficient sample size. Moreover, these two models demonstrated the lowest ROC AUC values in the external validation sample (0.53 and 0.38, respectively), indicating limited predictive power. For these reasons, these two models were excluded from the analysis of long-term effects.

### Polygenic profiling of older adults

To study long-term effects of genetic variants associated with different sports categories, an additional analysis was carried out in 2,914 older adults by comparing both the frequencies of genetic variants found significant in the GWASs (Supplementary Table S2) and the polygenic scores (Figure 5). Compared to the general population sample, older adults scored higher for coordination-based sports, cyclic sports, and game-oriented sports (Figures 5A–C) and, on average, significantly lower for combat sports (Figure 5D). Notably, sex had a fairly large weight in the model for combat sports; therefore, to address the effect of sex ratio imbalance, the data were visualized only for men in all three samples (Figure 5D).

**Figure 5:**
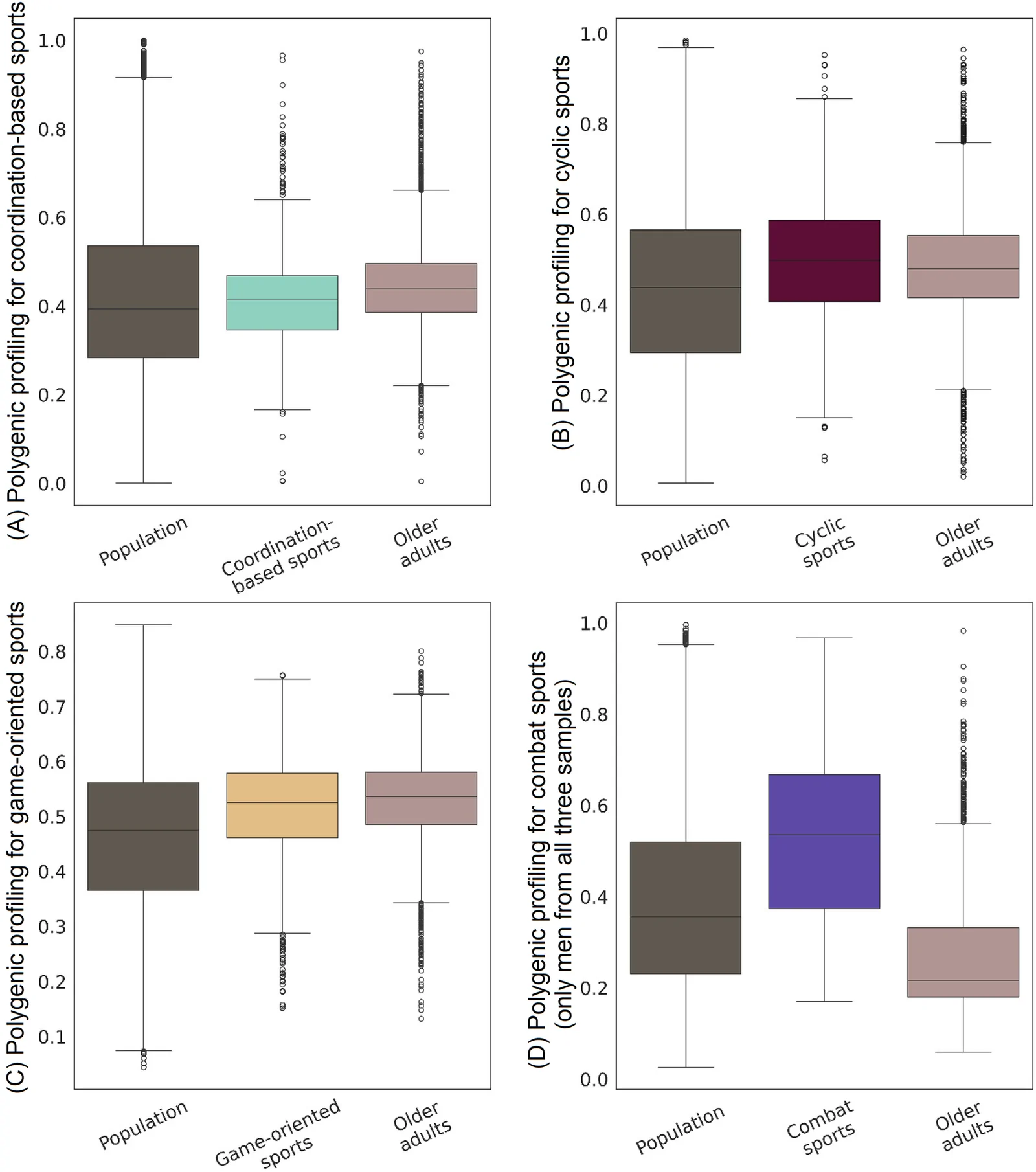
Polygenic profiling of participants from the sample, athletes from sports categories, and older adults. **(A)** Polygenic profiling for coordination-based sports. **(B)** Polygenic profiling for cyclic sports. **(C)** Polygenic profiling for game-oriented sports. **(D)** Polygenic profiling for combat sports (only men from all three samples).

To further examine the long-term effects of the detected genetic variants, the associations between polygenic scores and physical and clinical characteristics of older adults were analyzed (Table 5). Polygenic scores for coordination-based sports were negatively correlated with grip strengths and positively correlated with blood lipid levels, which is consistent with the data on grip strength and fatty mass obtained in the athletes. Other polygenic scores expectedly showed positive correlations with grip strength and a negative correlation with lipid levels. Older adults who scored higher for combat sports and cyclic sports also scored higher for functional and cognitive health assessed through the Short-Physical Performance Battery (SPPB) and the Mini Mental State Examination (MMSE). However, none of the polygenic scores were directly associated with frailty and sarcopenia.

**Table 5:** Correlations between polygenic scores and physical and clinical characteristics of older adults.

Characteristic	Coordination-based sports		Combat sports		Game-oriented sports		Cyclic sports	
	Coefficient	*p*-value*	Coefficient	*p*-value*	Coefficient	*p*-value*	Coefficient	*p*-value*
SPPB	−0.76	0.11	2.38	1.01*10^−8^	0.99	0.11	1.04	0.02
Frailty (men)	−0.48	0.77	−0.95	0.52	0.57	0.78	2.31	0.09
Frailty (women)	0.68	0.55	−0.56	0.63	−0.33	0.83	1.61	0.17
Barthel index	−3.25	0.46	20.50	1.19*10^−7^	14.02	0.02	16.15	5.59*10^−5^
Right-hand grip strength	−2.72	0.02	12.27	8.45*10^−33^	7.03	6.93*10^−6^	8.95	2.37*10^−17^
Left-hand grip strength	−2.04	0.07	12.00	3.38*10^−34^	7.63	3.37*10^−7^	9.02	4.06*10^−19^
SARC-F	0.42	0.32	−1.71	3.76*10^−6^	−0.81	0.15	−1.23	0.0013
Sarcopenia (men)	1.11	0.55	−1.16	0.47	−0.19	0.93	0.86	0.57
Sarcopenia (women)	−0.41	0.66	1.56	0.2	−0.66	0.62	−0.07	0.94
MMSE	−0.83	0.49	4.54	1.98*10^−5^	2.09	0.18	4.31	7.34*10^−5^
Triglycerides	0.29	0.003	−0.27	0.002	−0.18	0.17	−0.19	0.03
Total cholesterol	0.73	0.0006	−1.42	5.97*10^−14^	−0.65	0.02	−1.08	2.88*10^−8^
Free Т3	0.15	0.26	−0.29	0.01	−0.02	0.89	−0.02	0.87
Testosterone (men)	−2.20	0.48	0.04	0.99	−6.71	0.08	−4.15	0.15

* Adjusted for age, BMI, and multiple testing.

## Discussion

The following discussion presents preliminary hypotheses based solely on genomic locations of the identified variants and the functions of nearby genes. The mechanisms outlined here were not functionally validated in this study; their interpretations, therefore, should be considered merely hypothetical and intended to guide future research.

The study demonstrated an association between genetic variants and athlete status: the frequency of multiple variants differed significantly between professional athletes and non-athletes from the population sample. While each sports category possessed distinct genetic predictors, a shared set of variants was also observed across the categories. This finding is, most likely, attributable to certain biological effects the variants have, leading to the development of physical characteristics required for a particular sports category. The question is: What biological effects? To answer this question, we analyzed the functions of the genes that contained significant variants and identified a pool of biological processes that are significant for each sports category (Figure 6).

**Figure 6:**
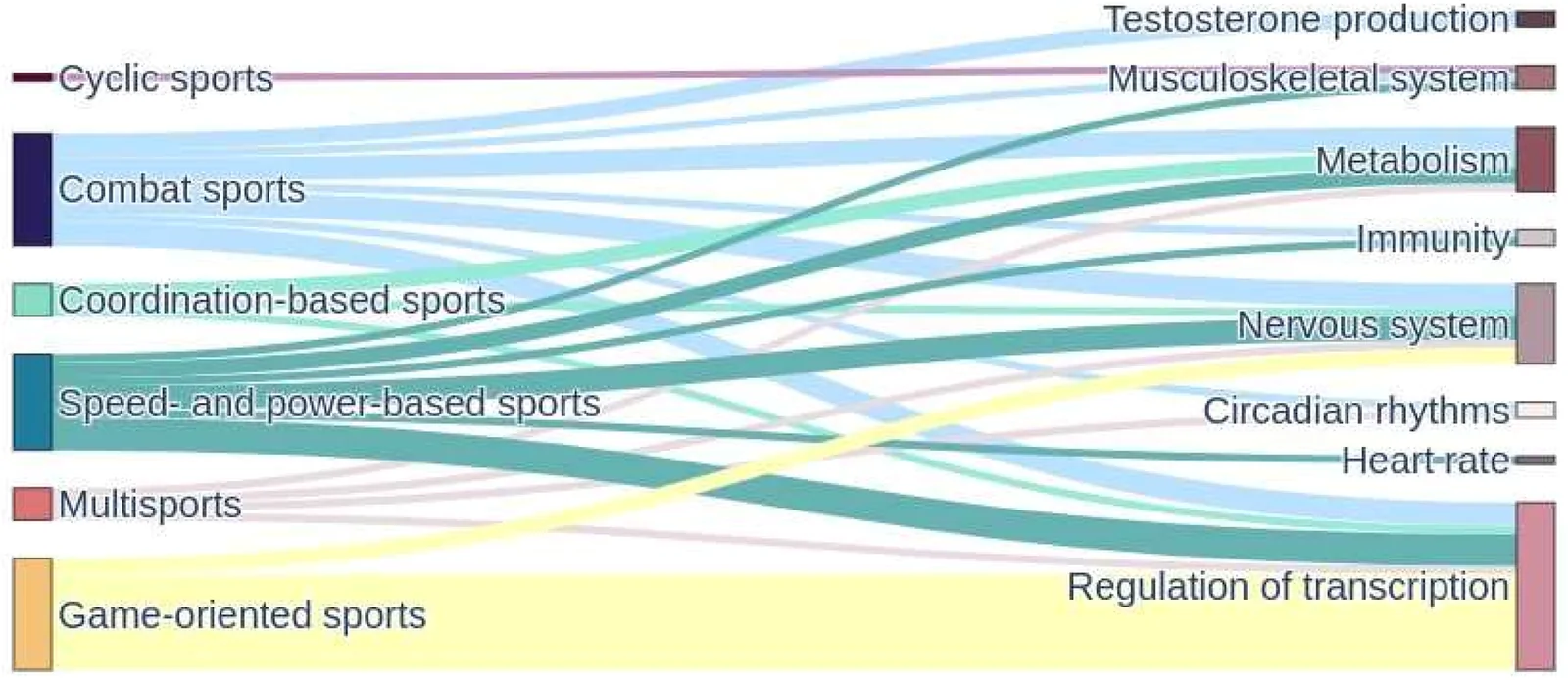
Functional annotation of significant GWAS findings based on the function of their respective genes.

Importantly, our findings demonstrate statistically significant differences in polygenic scores between broad sports categories and the control. However, these findings should not be interpreted as predictive indicators of an individual's athletic performance, competitive ranking, or career longevity. The models presented here are tools for group-level discrimination rather than predictive decision-making at the individual level. Any future application to talent selection would require prospective validation and must account for the multifactorial nature of performance. We therefore encourage a cautious interpretation of these findings as evidence of talent prediction or performance optimization.

The above diagram, along with the identification of the shared cluster of variants (Figure 2), indicated that there is a certain commonality across all athletes, associated, among other things, with the regulation of basic cellular processes, such as transcription and metabolism. All significant variants are located in intergenic or intron regions, i.e., they do not directly affect the structure of the encoded proteins, but they can affect the expression levels of specific genes. Moreover, most of the intergenic variants are actually located in the binding sites for transcription factors, which also highlights their potential regulatory role. For example, variants significant for multi-sports were located in the *TNRC6A* gene, involved in RNA interference (22), within the binding sites for transcription factors of the *CTCF* and *CTBP1* genes [proliferative activity (23–25)], the *HDAC2* and *PKNOX1* genes [transcription regulation during embryogenesis (26, 27)], and the *ARNT* gene (xenobiotic metabolism and transcription regulation in hypoxia) (28). The rs1039083409 and rs12577360 variants were significant for the game-oriented sports and combat sports. These variants are located in the recognition sites for transcription factors of the *CREB1* and *CLOCK* genes responsible for circadian rhythms (29–31). Through the temporal activation of the sympathetic nervous system and other mechanisms, circadian rhythms modulate the availability of carbohydrates and lipids throughout the day, affecting muscle activity (32, 33).

Many identified variants were associated with the development and functioning of the nervous system, such as rs2058218638 in the *LAMA1* gene, which was part of the shared cluster. This gene is involved in cerebellum development and movement coordination (34)—one of the most important skills for any athlete. This variant exhibited the greatest significance in the GWASs for game-oriented sports, coordination-based sports, cyclic sports, and combat sports. Variants in genes linked to the nervous system were also detected in the multi-sports category, such as *TGIF1*, involved in forebrain patterning (35). The rs144380919 variant from the same GWAS is part of the binding sites for the transcription factors of the *SCRT1* and *ATF3* genes involved in neuronal differentiation and cellular response to stress (36, 37). Three of the variants identified for speed- and power-based sports are located in the introns of the *DISC1FP1* [mood fluctuations (38)], *LAMB1* [nutrient supply to the brain (39)], and *CTNNA2* [synaptic plasticity (40)] genes. Interestingly, the intron of the *CTNNA2* gene contains the rs189583925 variant, which may be part of the binding site for the transcription factors *SMARCA4* and *GATA3* associated with proliferative activity (41), cholesterol levels (42), and parathyroid-mediated calcium metabolism (43).

Interestingly, only a small number of variants was associated with the organ system believed to be directly responsible for sports achievements—the musculoskeletal system. For example, variants in genes related to bone density were found in the coordination-based sports [CNBD1 (44)] and speed- and power-based sports [*DISC1FP1* (45) and *CTNNA2* (46)]. One of the variants detected in cyclic sports is located in the intron of the *TAFA5* gene, which activates osteoclast differentiation (47, 48). The *ZBTB40* gene (multi-sports) is associated with the regulation of osteoblast development (49). The activity of this gene can affect recovery from injuries such as bone fractures, which are common in cyclic sports, such as track and field. Interestingly, this variant is also part of the shared cluster, i.e., it is common for athletes across the sports categories.

Nevertheless, polygenic scores were correlated with certain anthropometric and physical characteristics, including the musculoskeletal system, such as grip strengths and muscle mass. Interestingly, the game-oriented sports, combat sports, and cyclic sports were correlated with increased muscle mass, while coordination-based sports were correlated with decreased muscle Notably, different types of muscle fibers and muscle characteristics provide for different types of physical activity. For example, athletes predominantly engaged in strength training are characterized by hypertrophy of fast-twitch muscle fibers (type IIA and IIX) (50, 51) that have a substantial capacity for glycolysis, i.e., they use the accumulated glycogen faster (52). In contrast, endurance athletes are more likely to have more developed slow-twitch muscle fibers (type I) (53), which produce energy more slowly but do so more efficiently due to high oxidative activity (52) and larger mitochondrial volume (54). With this efficient energy metabolism, the fibers often use fatty acids as an energy source and are capable of accumulating triglycerides (53). This fact may account for the positive correlation between lipid levels and adipose tissue mass in participants who scored higher for coordination-based sports, since athletes engaged in this sports category are more likely to train for endurance and, most likely, have a large proportion of type I fibers. The body girth measurements may also indirectly indicate a different pattern of muscle distribution. For example, sprinters and athletes predominantly engaged in strength training are known to have larger muscles on their extremities compared to endurance athletes (55). This observation is consistent with our findings on the positive correlation between shoulder and lower leg girth measurements and polygenic scores for combat sports, game-oriented sports, and cyclic sports and the negative correlation with polygenic scores for coordination-based sports. Based on the findings of this study, we cannot unequivocally state that there is a cause-and-effect relationship between the genetic variants underlying polygenic scores and the examined physical characteristics. Further studies could shed light on the nature of these relationships.

Due to certain similarities, athletes in cyclic sports and game-oriented sports share common physical characteristics. Interestingly, polygenic scores for coordination-based sports were correlated with the scores for cyclic sports and game-oriented sports; however, physical characteristics of athletes from coordination-based sports differed due to the predominance of endurance training. Polygenic scores for combat sports, which share physical demands with other power-based sports, were weakly but negatively correlated with other scores. Notably, in older adults, the polygenic score for combat sports was lower than the population average, indicating a negative association with longevity. In combat sports athletes, several variants were identified in genes involved in steroid metabolism (e.g., AKR1C3) (56, 57), yet no significant association was found between the combat sports polygenic score and testosterone levels in older adults. Therefore, the biological basis of the negative correlation between the combat sports polygenic score and exceptional longevity remains unclear and warrants further investigation. Given the strictly preliminary nature of our findings, we emphasize that the presented genetic profiling framework should not be used for talent selection among children or adolescent athletes. Our results do not provide sufficient evidence to justify any practical application of the framework in matching individuals to specific sport disciplines. Furthermore, genomic markers alone are not definitive predictors of an athlete's success in any specific sport, as athletic performance is influenced by a wide range of environmental, psychological, and training-related factors.

If there is a mismatch between a child's aspirations and the outcomes of genetic tests, they may face various psychological challenges. For example, they might choose to discontinue their training if their genotype is not aligned with their desired sport. Alternatively, they may develop an inflated sense of confidence in their abilities, which could lead to decreased motivation for training and a subsequent decline in performance. Given these concerns, it is crucial to approach the implementation of sports genomics with caution. Recommendations should be clear, balanced, and primarily intended for coaches. These guidelines should go beyond a straightforward interpretation of the results, as genomic markers alone cannot determine an athlete's success in any specific sport. The multifaceted nature of athletic performance is influenced not only by genetic factors but also by a wide range of environmental factors.

### Limitations

This study has several limitations that should be considered when interpreting its findings. First, the cross-sectional design evaluates athletes, population controls, and older adults at a single point in time, which allows for association analyses but precludes a prospective prediction of athletic success, training response, career trajectory, or lifespan. Second, the six genome-wide association studies are exploratory case–control comparisons that share a single common control group. Consequently, they are not independent discovery experiments, and the standard genome-wide significance threshold of 5 × 10^−8^ is less conservative than it would be with independent controls; shared confounding factors may have affected all six models. Third, the small sample sizes of several categories, notably speed- and power-based sports (*n* = 146) and multi-sports (*n* = 102), may have underpowered the analyses; hence, the findings pertaining to these specific cohorts should be treated as purely hypothesis-generating. Fourth, while the polygenic score models were validated in an independent external sample not used in the GWAS or feature selection, the external validation set was relatively modest in size and drawn from a similar population. Therefore, the reported AUC values, although free of the optimistic bias inherent in in-sample evaluation, still require confirmation in larger, more diverse cohorts before considering generalizability. Furthermore, the obtained scores were not calibrated and should not be used for individual-level estimation. Fifth, the sports categories are formed based on a training- and dominant physical trait-based classification (pedagogical perspective) rather than biomechanical similarities. Therefore, the intra-category physiological heterogeneity and between-category overlap may reduce the specificity of the observed signals and model performance. Finally, no information on competition level, international ranking, objective performance metrics, or career duration was available, which further limits any performance-related interpretation.

For these reasons, the results should be interpreted as cross-sectional genetic associations that distinguish broad athlete groups from the population control at a group level, rather than as evidence for individualized prediction of athletic achievement or longevity. External validation in independent cohorts, coupled with calibration and objective performance outcomes, would be required before any practical application.

## Conclusion

The study identified genetic variants associated with athlete status across various sports categories. The findings indicate that while certain genetic traits are common among different sports categories, there are also distinct traits unique to each one of them. These insights contribute to a better understanding of the genetic architecture of athlete status and may generate hypotheses for future longitudinal studies. The polygenic score models developed here demonstrate statistical discrimination between broad athlete groups and population controls, but current evidence does not support any practical application in talent identification or performance prediction.

The results obtained have both practical and fundamental value. Although the link between physical activity and increased life expectancy is well-established, the findings suggest that this correlation goes beyond the benefits associated with a healthy lifestyle and training programs. It appears that the innate genetic predisposition toward certain physical traits may also play a role, both in athletes and older adults.

Given the exploratory nature of this study, we caution against using genomic data for sport selection or training program design. Athletic performance is multifactorial, and genetic information represents only one of many contributing factors. Future research with prospective designs and objective performance metrics is needed to determine whether polygenic scores have any practical utility in sport science.

## Data Availability

The individual-level whole-genome sequencing data analyzed in this study are not publicly available. These data constitute personal genetic information of identifiable human participants, and their public release is restricted by the terms of the informed consent approved by the local ethics committee and by the Russian legislation on the protection of personal data. All variants that reached genome-wide significance in the six association analyses, together with their genomic coordinates, alleles, effect sizes, allele frequencies and p-values, are reported in full in Table 3 and Supplementary Table S2 of this article. Population-level variant frequencies for the Russian population are openly available in the FMBA population variant frequency database (https://gdbpop.nir.cspfmba.ru/). The genome-wide summary statistics for all six analyses will be deposited in a public repository and made openly available once the corresponding institutional authorization for their release has been obtained; until then, they may be requested from the corresponding author. Further enquiries can be directed to the corresponding author (mterekhov@cspfmba.ru).
