# Supplementary material for: GWAS-based polygenic profiling across broad sport categories: shared and category-specific genetic associations with athlete status and polygenic differences from population controls
Source: Front Sports Act Living. 2026 Sep 30;8:1813231. doi: 10.3389/fspor.2026.1813231 (PMC13663180; doi:10.3389/fspor.2026.1813231)
Supplement: Supplementary file 1 [file Datasheet1.pdf]

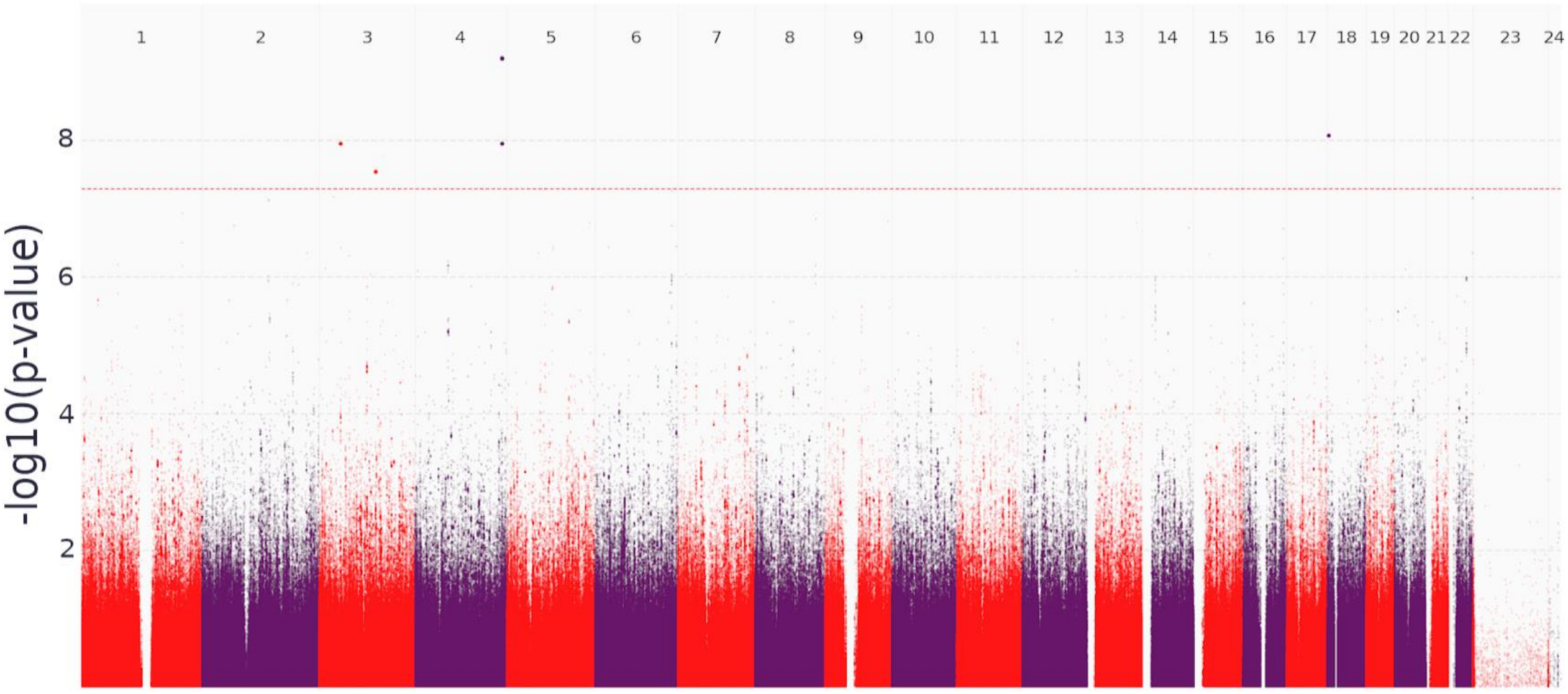

Figure S1. GWAS findings for multi-sports.

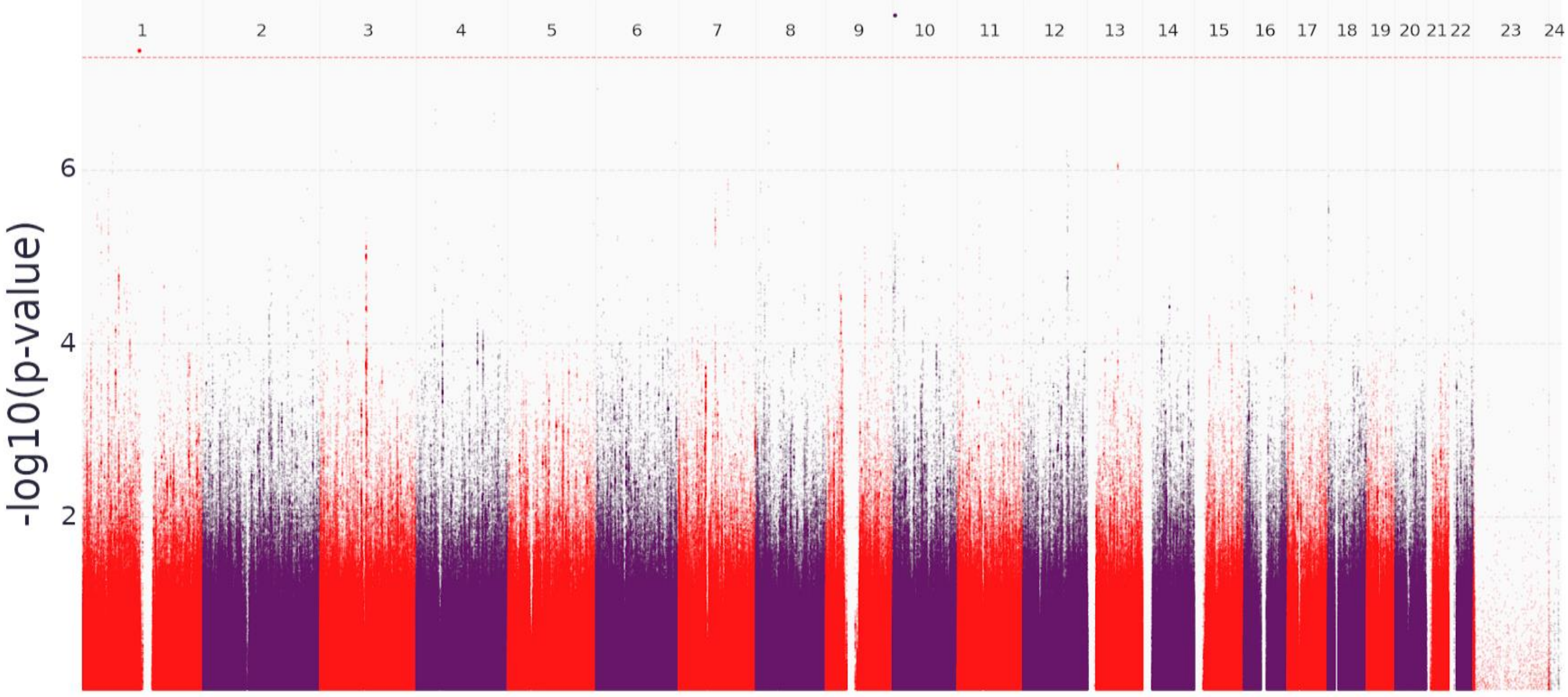

Figure S2. GWAS findings for combat sports.

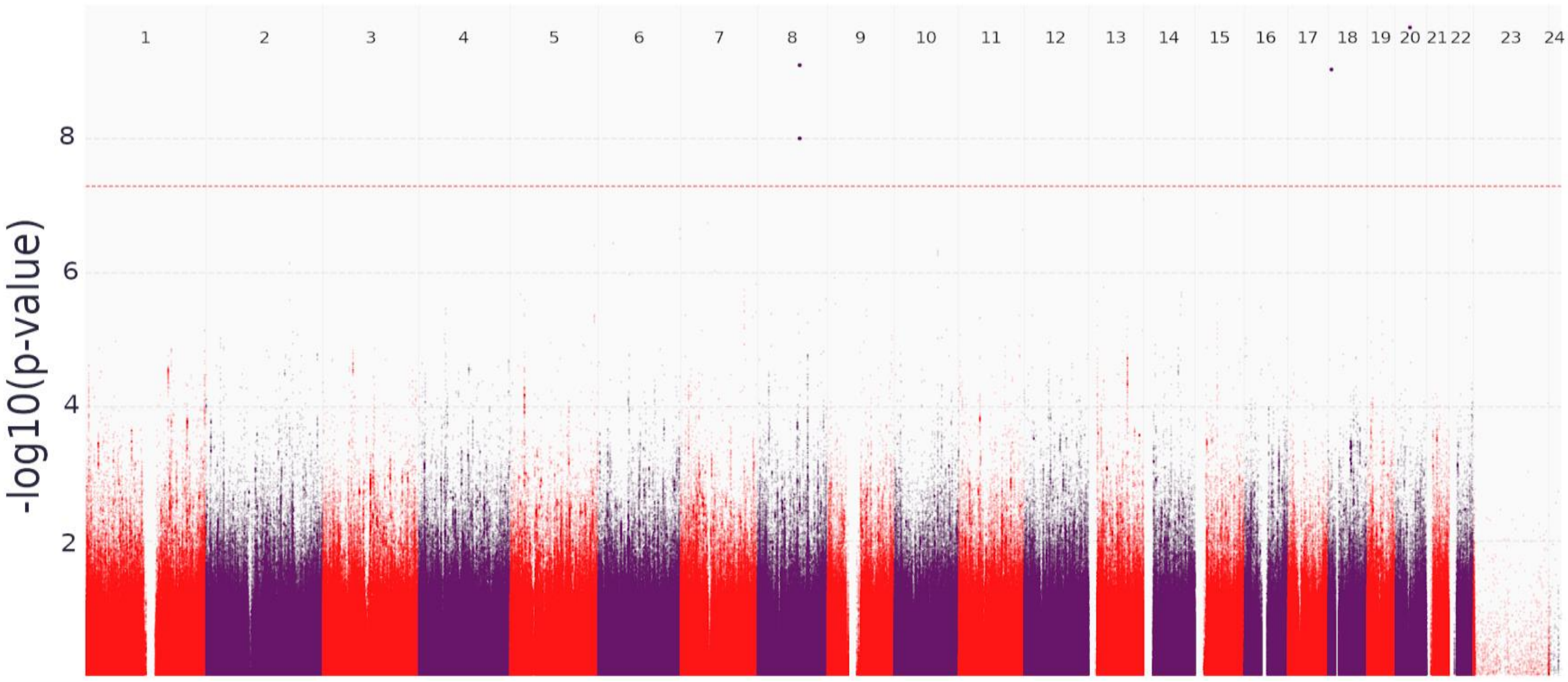

Figure S3. GWAS findings for coordination-based sports.

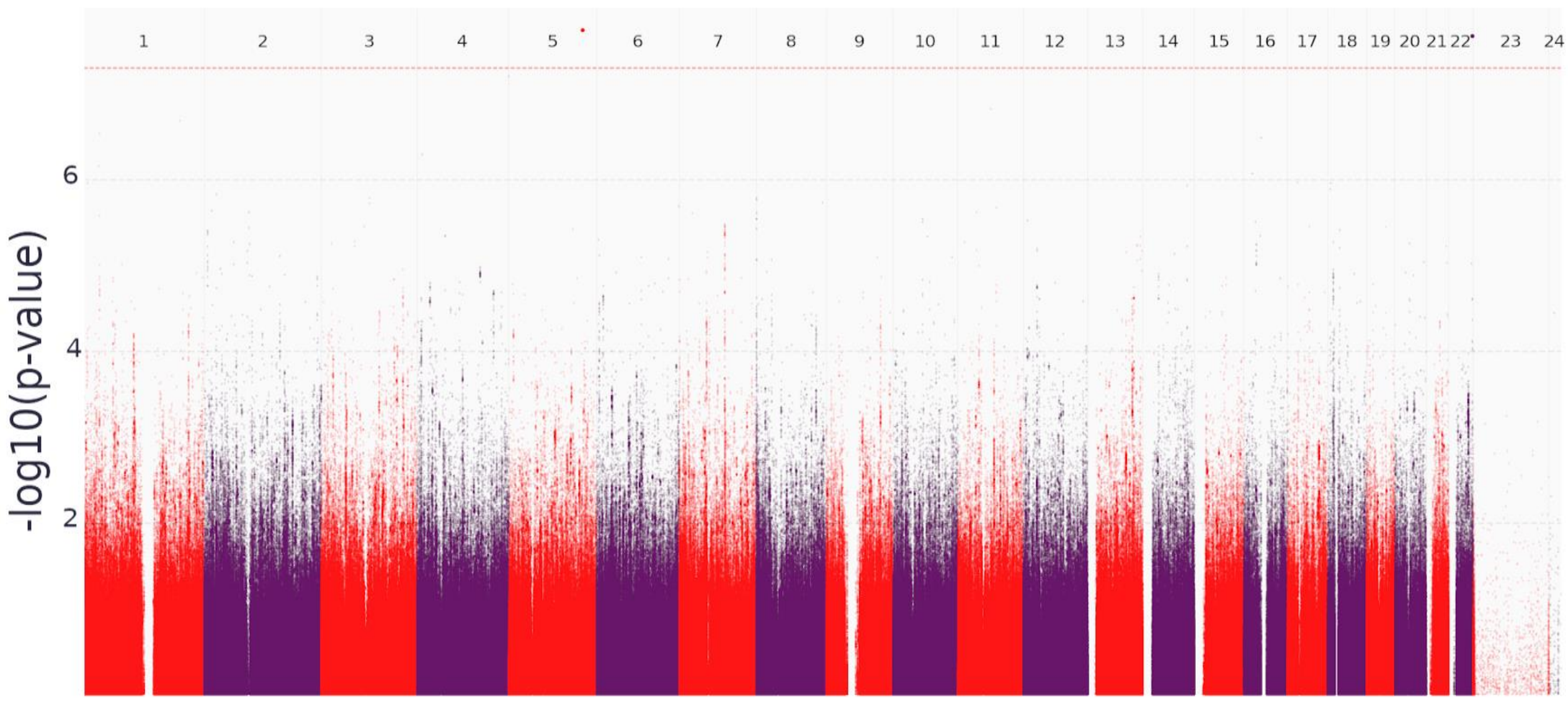

Figure S4. GWAS findings for cycling sports.

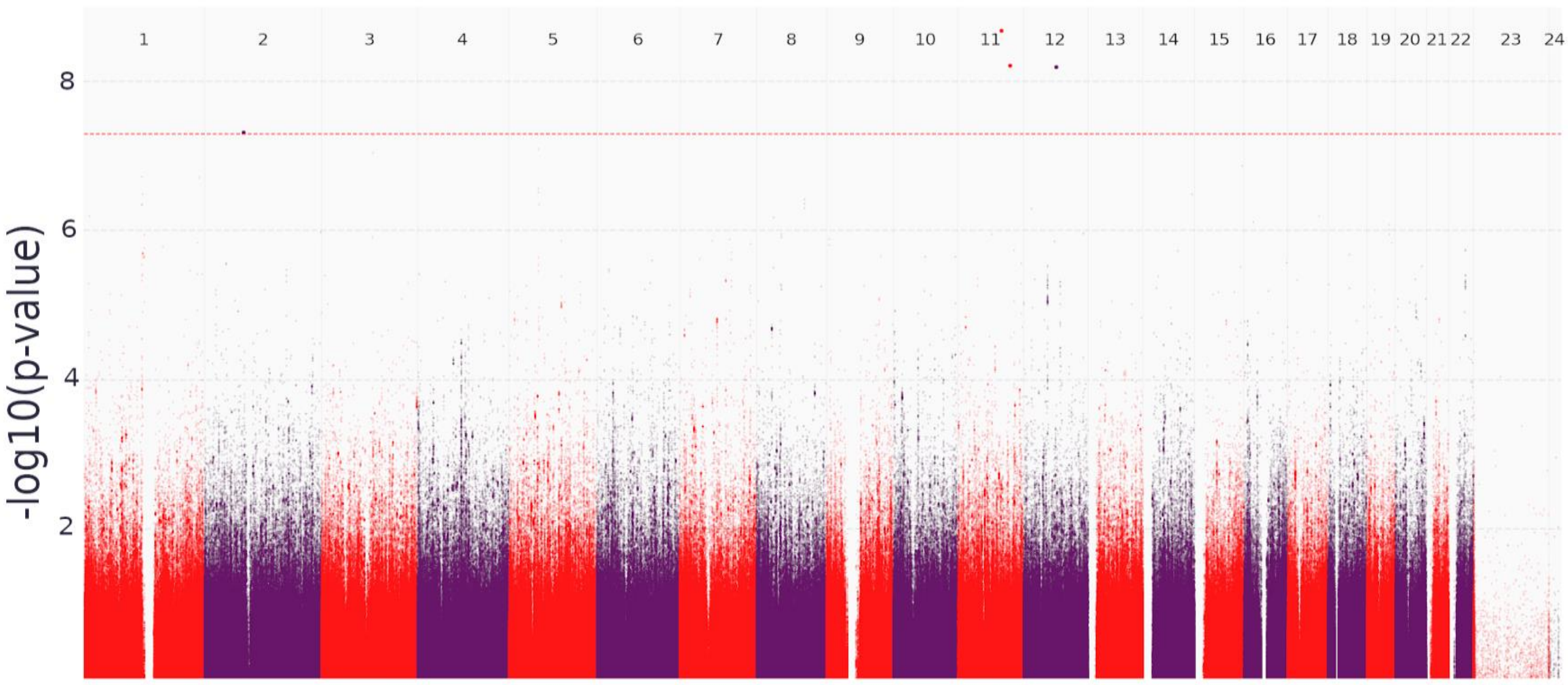

Figure S5. GWAS findings for speed- and power-based sports.

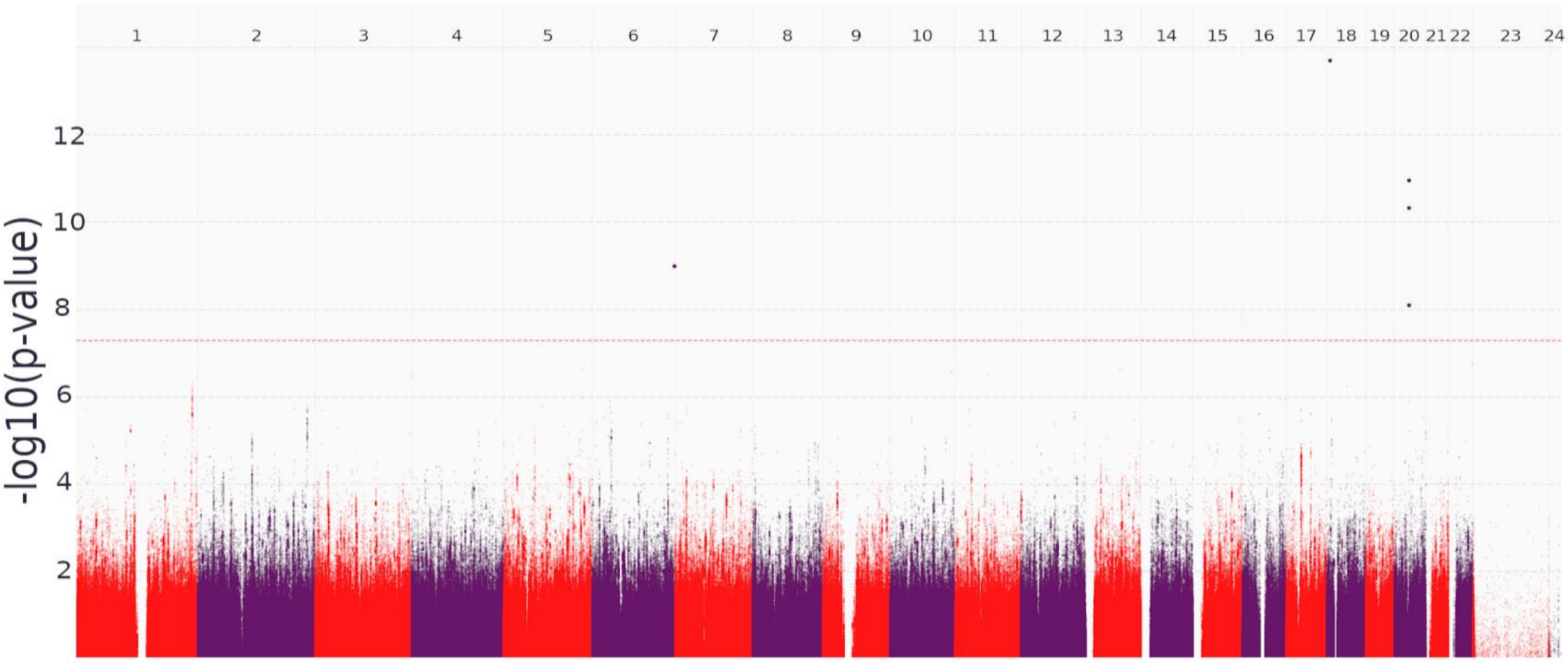

Figure S6. GWAS findings for game-oriented sports.

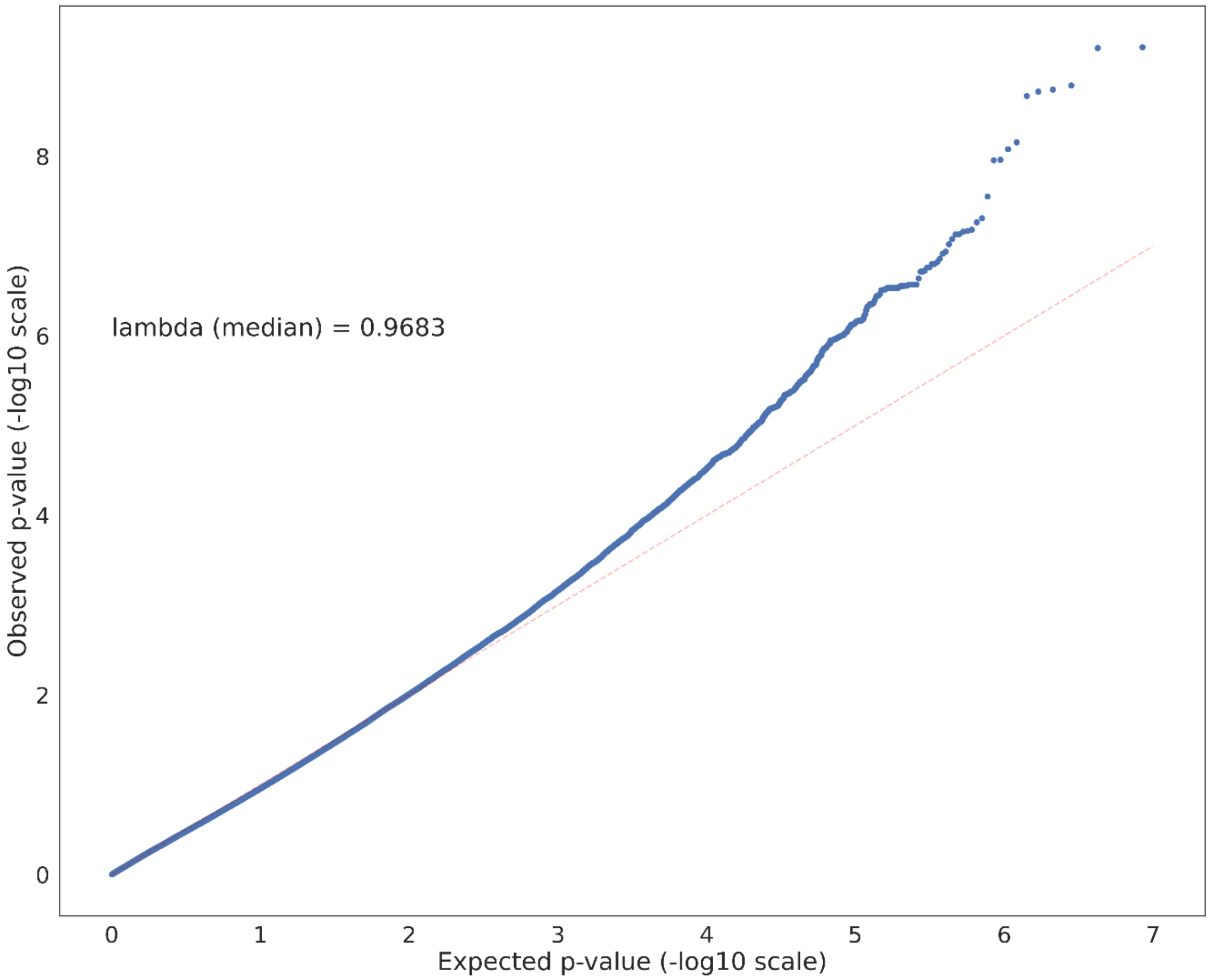

Figure S7. QQ-plot for multi-sports.

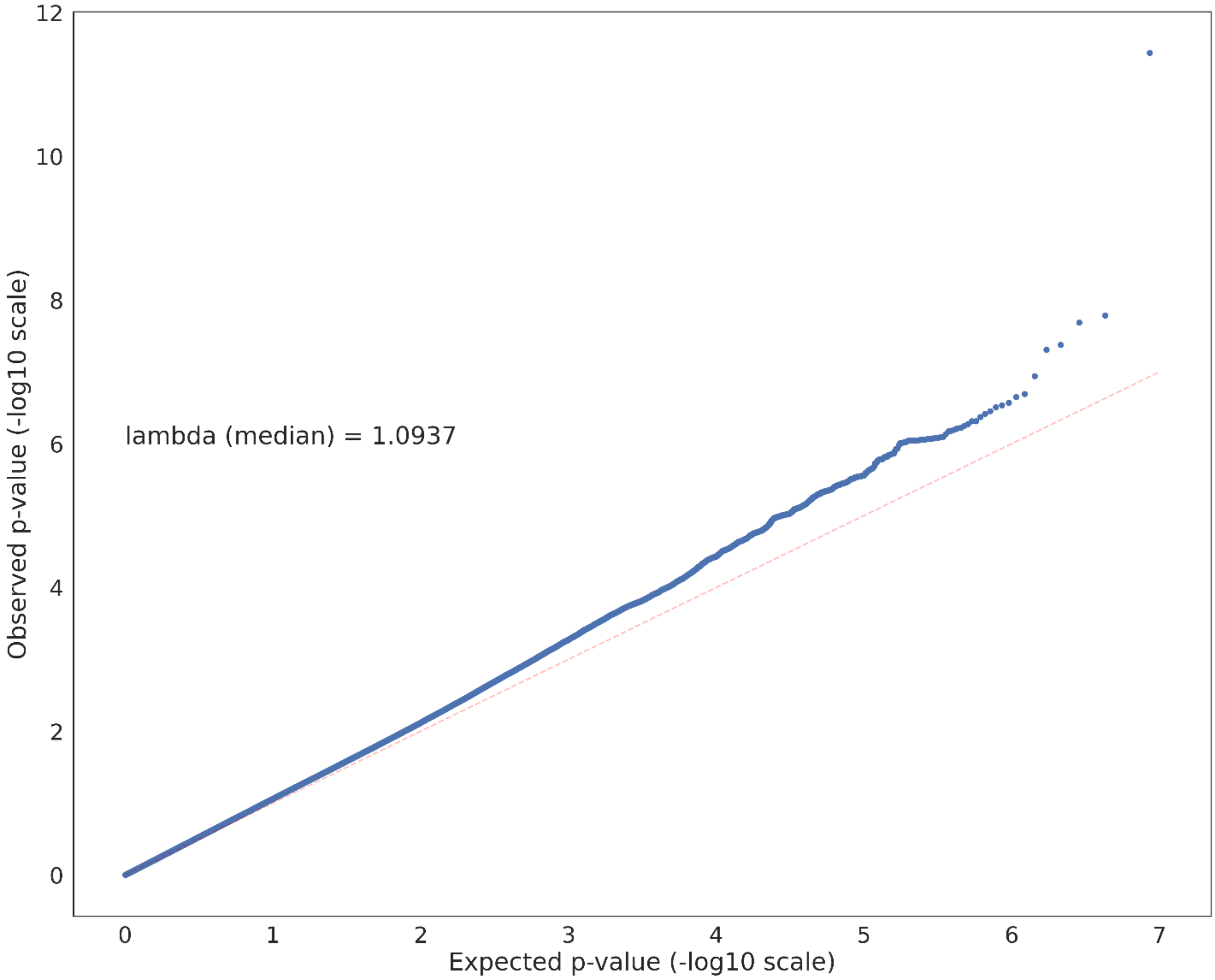

Figure S8. QQ-plot for combat sports.

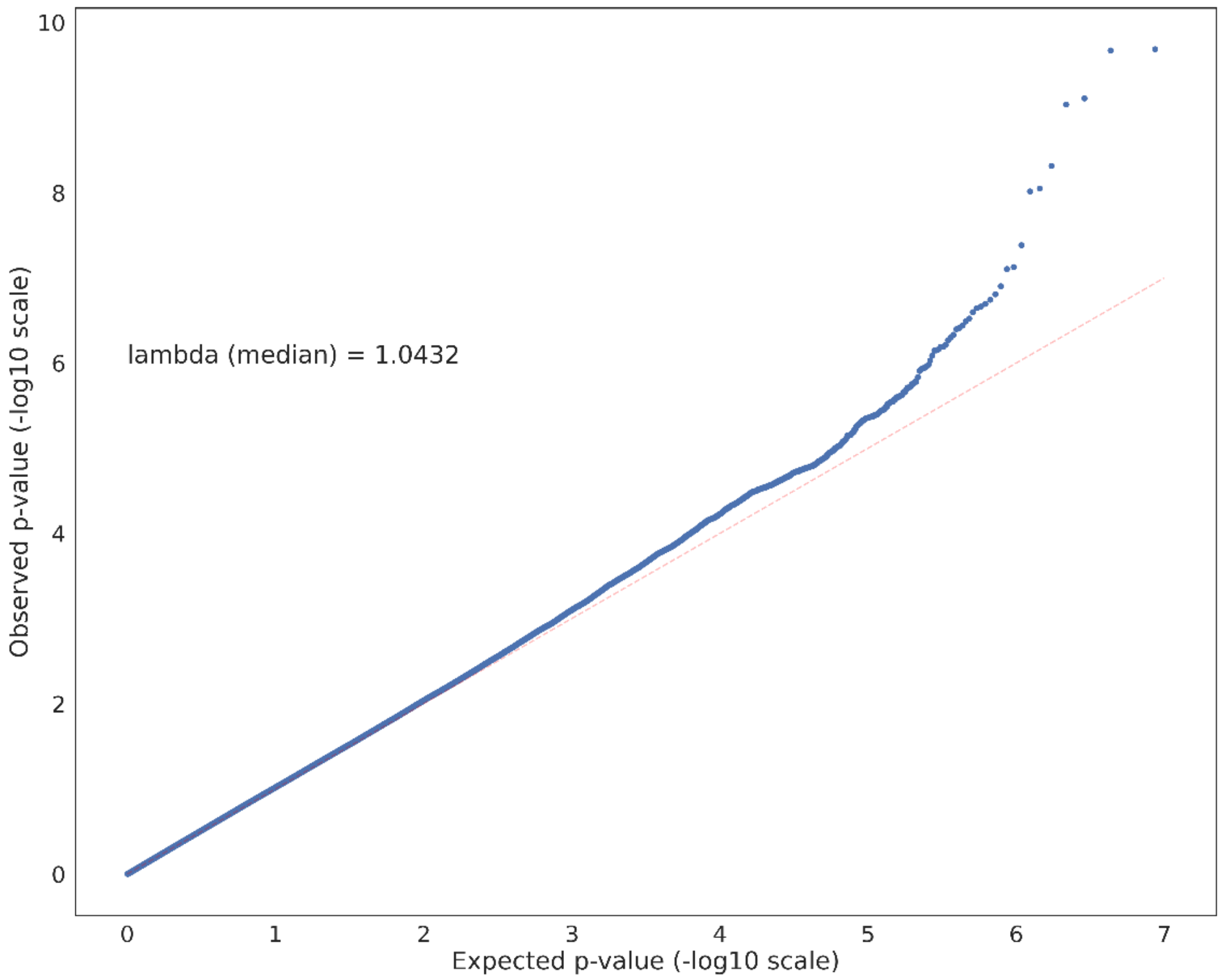

Figure S9. QQ-plot for coordination-based sports.

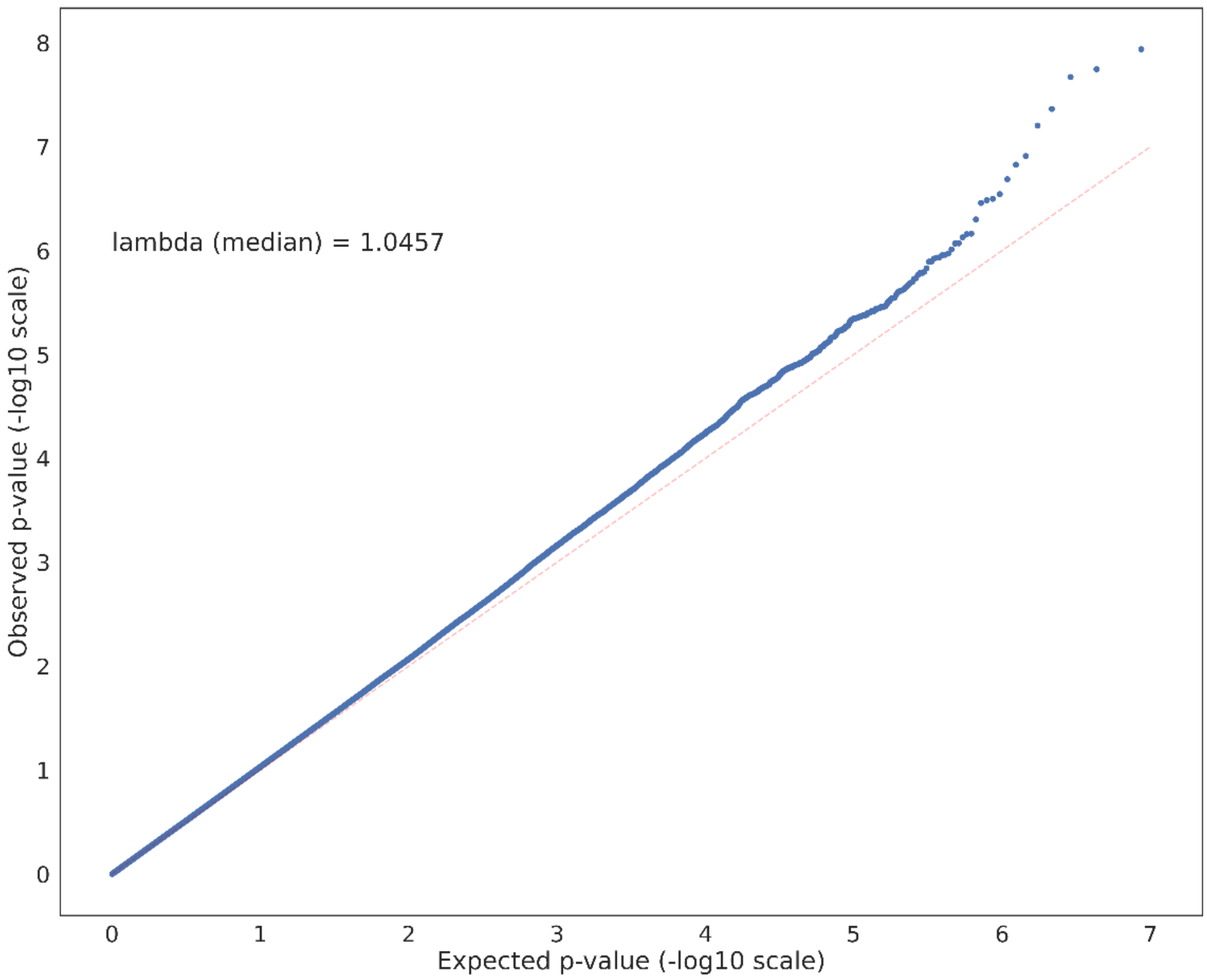

Figure S10. QQ-plot for cycling sports.

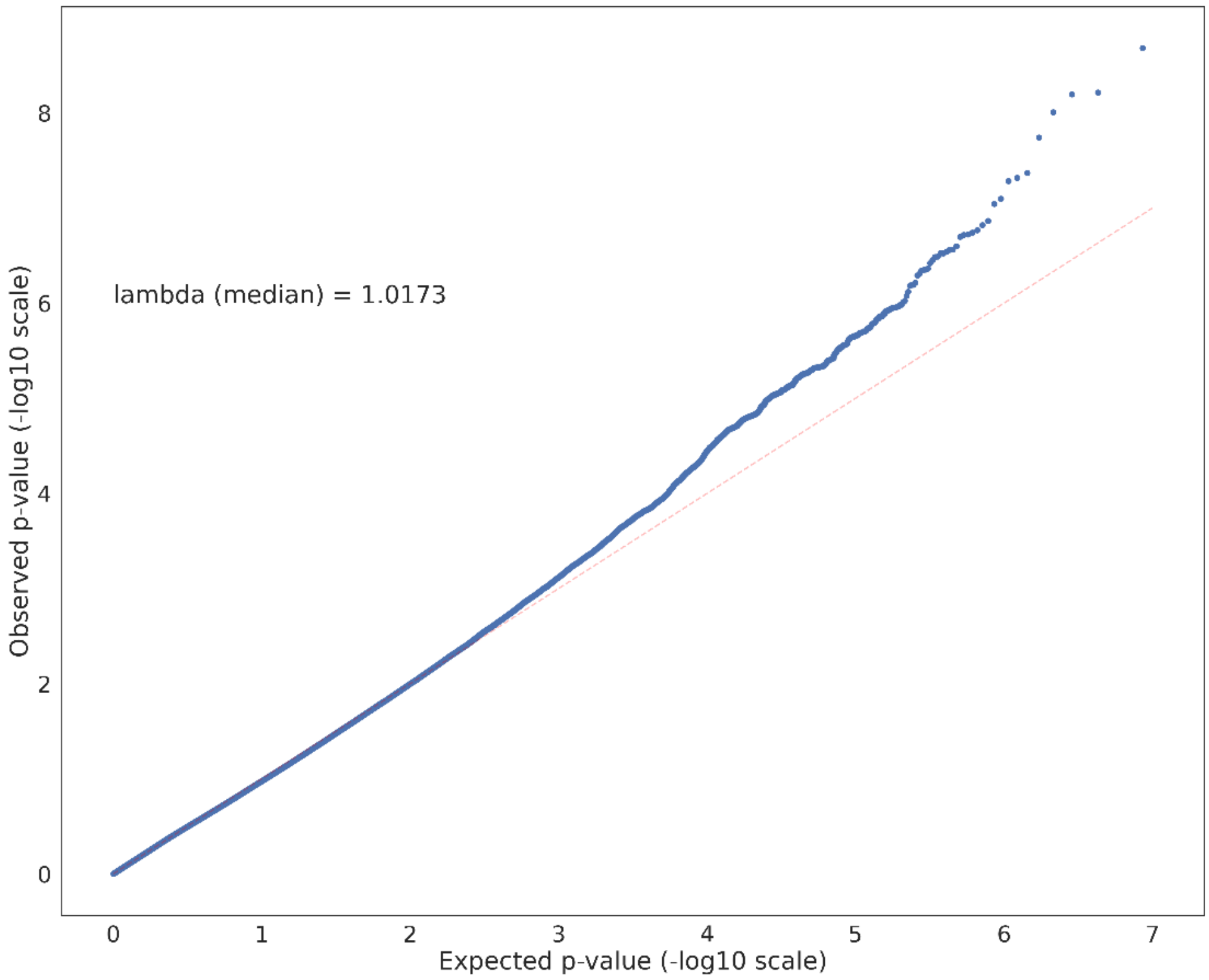

Figure S11. QQ-plot for speed- and power-based sports.

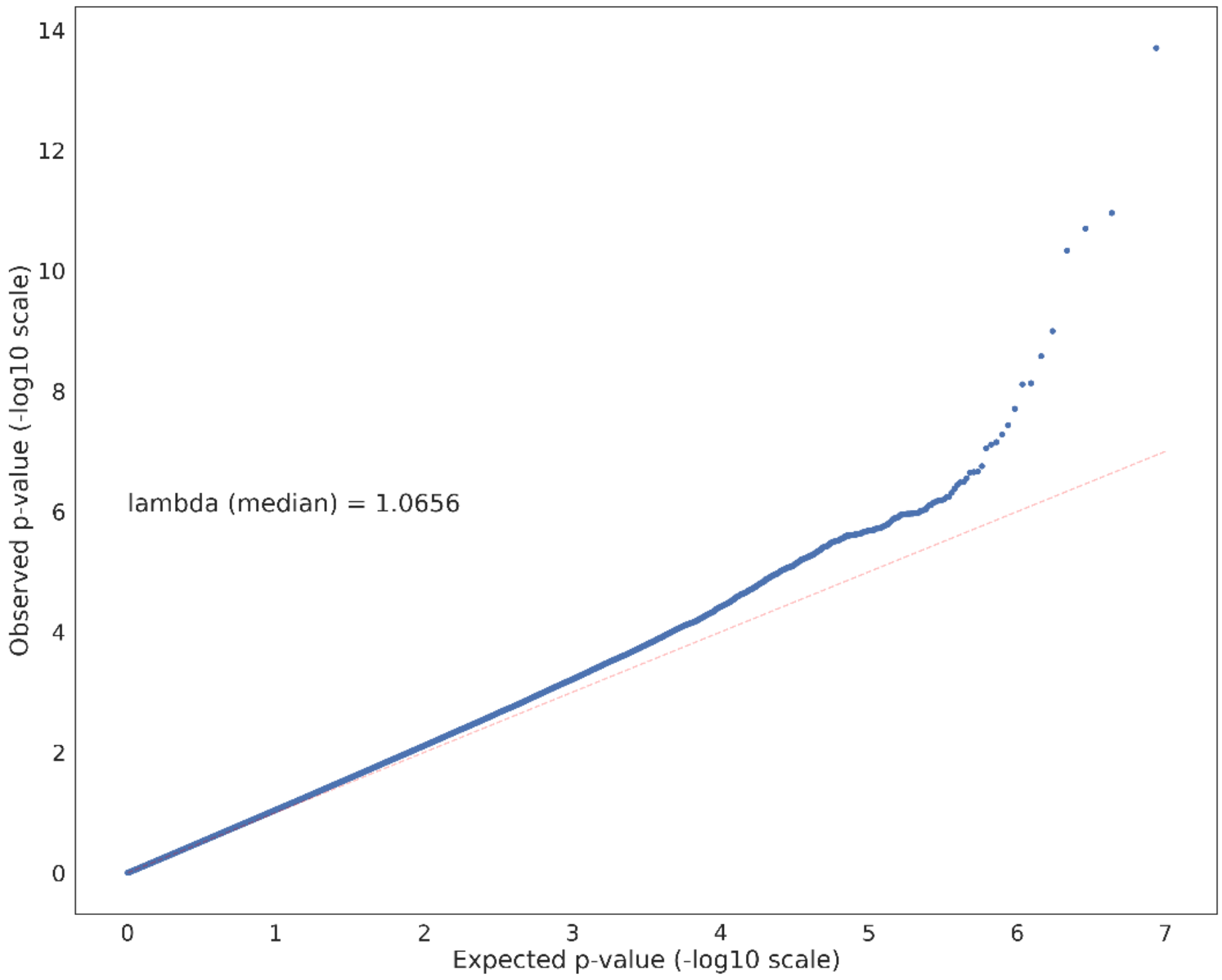

Figure S12. QQ-plot for game-oriented sports.

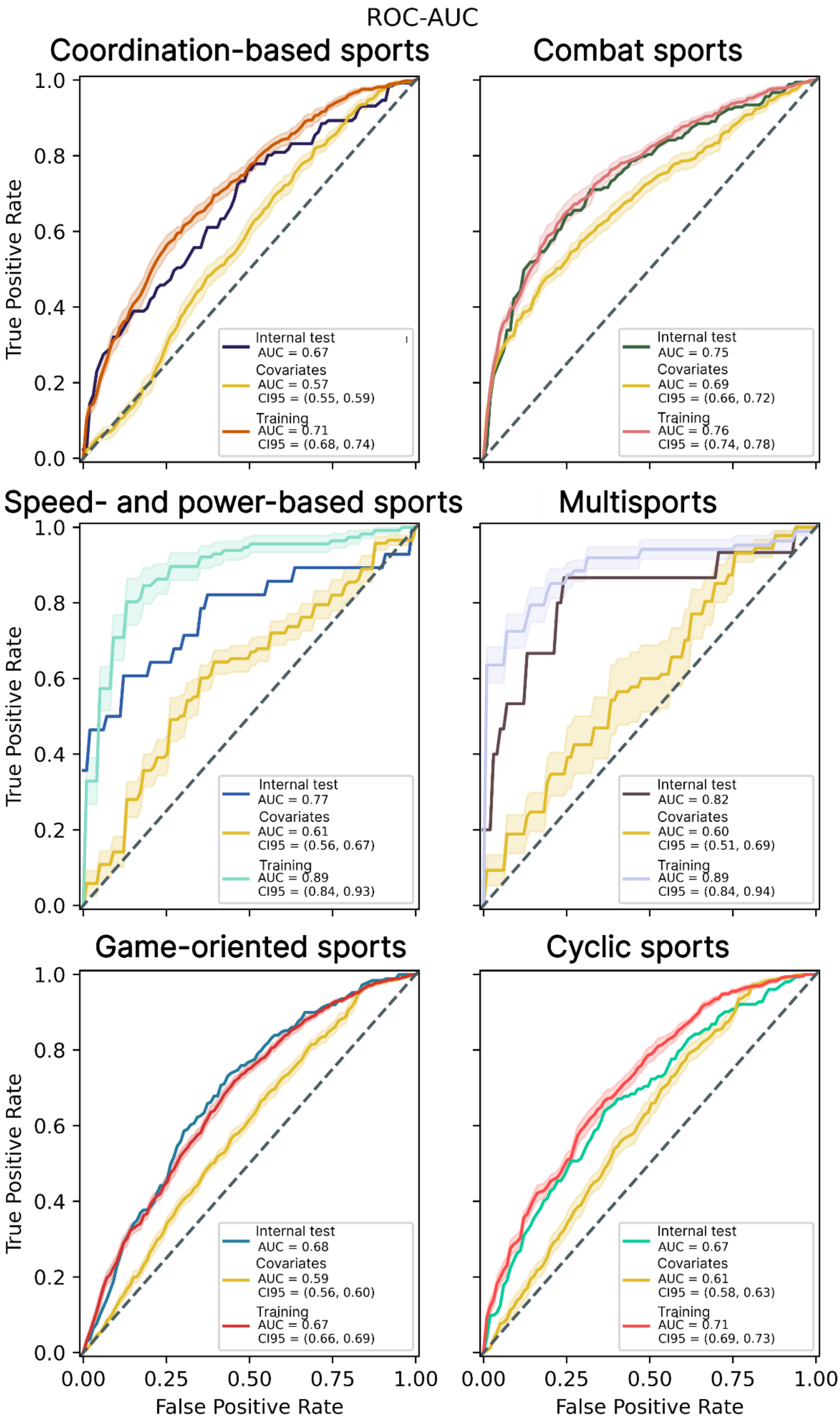

Figure S13. Model training and testing results.
