# Supplementary material for: GWAS-based polygenic profiling across broad sport categories: shared and category-specific genetic associations with athlete status and polygenic differences from population controls
Source: Front Sports Act Living. 2026 Sep 30;8:1813231. doi: 10.3389/fspor.2026.1813231 (PMC13663180; doi:10.3389/fspor.2026.1813231)
Supplement: Supplementary file 2 [file Datasheet2.docx]

**Supplementary methods. Samples filtration details.**

All sequenced samples were screened for cross-contamination (i.e., the presence of another individual’s DNA). Contamination levels were assessed using the allele ratio (AR) metric, as described by Dallavilla et al. [Dallavilla T. et al. A fast, reliable and easy method to detect within-species DNA contamination. Acta Bio Medica Atenei Parm. 2020; 91(Suppl 13): e2020019]. Using bcftools v1.21 (as of September 12, 2024) [29], we calculated the ratio of reads supporting one of the two alleles at biallelic positions relative to the total read count at that position, along with associated metrics. The applicability of this approach was demonstrated in a study by our colleagues, Feliz et al. [Feliz N.V. et al. Experimental Evaluation of the Possibility to Detect Cross-Contaminated DNA Samples Based on Genetic Data. Russ. J. Genet. 2023; 59(7): 719–728]. Samples with high contamination levels were excluded from further analysis.

For each sample, we also bioinformatically determined the Rh factor (based on RHD gene coverage) and sex (using DRAGEN’s built-in "Ploidy estimator" tool). These results were cross-checked with the information provided in the patient questionnaires. Discrepancies between the bioinformatics analysis and the questionnaire data prompted further investigation and, in some cases, led to the exclusion of the sample from the database.

We screened all samples for duplicates using PICARD CrosscheckFingerprints v2.26.11. When duplicate samples were identified, we cross-referenced the donor questionnaires. If the sequenced samples were confirmed to originate from the same donor, only the sample with the highest quality metrics (average alignment coverage over the genome ≥ 30x, GC content 38–40%, mapped reads >98%, duplicated reads ≤ 12%) was retained in the database. If the questionnaire information did not match, both samples were excluded.

Additionally, we calculated relatedness among samples using the kinship coefficient [Manichaikul A. et al. Robust relationship inference in genome-wide association studies. Bioinformatics. 2010; 26(22): 2867–2873]. For this calculation, we used the "FP_SNPs.txt" variant set from the GRAF software [Jin Y. et al. GRAF-pop: A Fast Distance-Based Method To Infer Subject Ancestry from Multiple GenotypeDatasets Without Principal Components Analysis. G3 Genes|Genomes|Genetics. 2019; 9(8): 2447–2461]. This set includes 10,000 genetic variants and is designed to identify duplicate samples and relatedness in large datasets [Jin Y. et al. Quickly identifying identical and closely related subjects in large databases using genotype data. PLoS ONE. 2017; 12(6): e0179106]. Pairwise calculations were performed for all sequenced samples. If a pair of samples showed a kinship coefficient exceeding the threshold for close relatedness (kinship > 0.177, corresponding to first- or second-degree relatives), only one sample from the pair was retained in the analysis. The sample with the highest sequencing quality metrics.

Thus, only samples meeting the following criteria were included in the analysis:

• No contamination with another individual’s DNA,

• Bioinformatics results (sex, Rh factor, relatedness) consistent with questionnaire data,

• No duplicates.
