# Supplementary material for: GWAS-based polygenic profiling across broad sport categories: shared and category-specific genetic associations with athlete status and polygenic differences from population controls
Source: Front Sports Act Living. 2026 Sep 30;8:1813231. doi: 10.3389/fspor.2026.1813231 (PMC13663180; doi:10.3389/fspor.2026.1813231)
Supplement: Supplementary file 3 [file Datasheet3.docx]

**Table S1 (Part 1).** Main anthropometric characteristics of athletes

| **Characteristic** | **Coordination-based sports** | | | **Combat sports** | | | **Speed- and power-based sports** | | |
| --- | --- | --- | --- | --- | --- | --- | --- | --- | --- |
|  | **Median and interquartile range**  **(Ме [Q1, Q3])** | **Regression coefficient** | **P-value** | **Median and interquartile range**  **(Ме [Q1, Q3])** | **Regression coefficient** | **P-value** | **Median and interquartile range**  **(Ме [Q1, Q3])** | **Regression coefficient** | **P-value** |
| BSA | *1.66 [1.49, 1.80]* | *-1.9* | *4.8×10^-8^* | *1.76 [1.61. 1.96]* | *-2.62* | *1.1×10^-16^* | *1.93 [1.73. 2.14]* | *-1.59* | *0.29* |
| Height, standing | *167.9 [161, 175]* | *-0.04* | *6.4×10^-12^* | *170.5 [164. 179]* | *-0.046* | *8.8×10^-19^* | *173.0 [166. 180]* | *-0.04* | *0.009* |
| Righ forearm girth, flexed | *30.0 [27.0, 32.0]* | *-0.1* | *0.004* | *33.0 [29.9. 36.0]* | *0.01* | *1* | *36.0 [32.5. 40.0]* | *0.02* | *1* |
| Left shin girth | *36.0 [34.0, 38.0]* | *-0.05* | *1* | *37.0 [35.0. 40.0]* | *-0.101* | *0.0004* | *40.0 [37.0. 43.0]* | *-0.004* | *1* |
| Right thigh girth | *55.0 [51.0, 59.0]* | *-0.04* | *0.32* | *57.0 [53.0. 61.0]* | *-0.128* | *6.5×10^-17^* | *64.0 [59.0. 68.0]* | *0.09* | *0.26* |
| Right forearm girth, relaxed | *27.0 [25.0, 30.0]* | *-0.1* | *0.028* | *30.0 [27.0. 33.0]* | *-0.015* | *1* | *33.0 [29.5. 37.0]* | *-0.003* | *1* |
| Left-hand grip | *34.0 [27.0, 43.5]* | *-0.03* | *4.6×10^-5^* | *41.5 [33.5. 50.5]* | *-0.01* | *1* | *47.5 [39.5. 60.5]* | *0.045* | *0.03* |
| Weist girth | *70.0 [65.0, 75.0]* | *-0.06* | *0.0004* | *74.0 [69.0. 81.0]* | *-0.043* | *0.05* | *82.0 [74.0. 90.0]* | *-0.07* | *0.39* |
| Right shin girth | *36.0 [34.0, 38.0]* | *-0.06* | *0.351* | *37.0 [35.0. 40.0]* | *-0.093* | *0.001* | *40.0 [37.0. 43.0]* | *0.014* | *1* |
| Neck girth | *35.0 [33.0, 37.0]* | *-0.02* | *1* | *37.8 [35.0. 41.0]* | *0.07* | *0.018* | *39.0 [36.0. 44.0]* | *0.084* | *1* |
| Righ-hand grip strength | *35.5 [28.0, 45.0]* | *-0.03* | *7.4×10^-5^* | *43.0 [35.0. 52.0]* | *-0.017* | *0.353* | *49.7 [40.2. 60.1]* | *0.03* | *0.42* |
| Chest girth during exhalation | *86.0 [80.0. 91.0]* | *-0.03* | *0.02* | *91.0 [85.0. 99.0]* | *0.003* | *1* | *97.0 [89.5. 108.0]* | *-0.019* | *1* |
| Left thigh girth | *55.0 [51.0. 58.0]* | *-0.05* | *0.02* | *57.0 [53.0. 61.0]* | *-0.12* | *7.7×10^-15^* | *63.0 [58.5. 68.0]* | *0.072* | *1* |
| Adipose tissue percentage | *13.3 [8.1. 18.2]* | *0.02* | *1* | *11.8 [8.3. 17.1]* | *-0.035* | *0.195* | *15.2 [11.2. 21.9]* | *-0.06* | *0.376* |
| Visceral fat percentage | *22.5 [5.0. 41.35]* | *0.01* | *0.149* | *18.8 [3.25. 43.2]* | *-0.005* | *1* | *34.7 [6.5. 67.4]* | *0.001* | *1* |
| Muscle mass | *27.9 [23.3. 34.15]* | *-0.08* | *2.9×10^-9^* | *32.3 [27.4. 39.6]* | *-0.05* | *8.3×10^-6^* | *37.1 [30.1. 45.3]* | *-0.001* | *1* |
| Hip-to-waist ratio | *0.8 [0.77. 0.82]* | *-1.09* | *1* | *0.81 [0.78. 0.85]* | *-0.13* | *1* | *0.83 [0.79. 0.88]* | *-6.54* | *0.02* |
| Fatty mass | *7.4 [4.4. 10.9]* | *0.003* | *1* | *8.10 [5.10. 12.20]* | *-0.05* | *0.026* | *11.9 [8.2. 18.0]* | *-0.11* | *3.3×10^-5^* |

*p-value obtained in regression analysis, with sports category as case. other participants as control, and sex, age, and BMI.

**Table S1 (Part 2).** Main anthropometric characteristics of athletes

| **Characterisic** | **Multi-sports** | | | **Game-oriented sports** | | | **Cycling sports** | | |
| --- | --- | --- | --- | --- | --- | --- | --- | --- | --- |
|  | **Median and interquartile range**  **(Ме [Q1, Q3])** | **Regression coefficient** | **P-value** | **Median and interquartile range**  **(Ме [Q1, Q3])** | **Regression coefficient** | **P-value** | **Median and interquartile range**  **(Ме [Q1, Q3])** | **Regression coefficient** | **P-value** |
| BSA | *1.77 [1.63, 1.89]* | *1.195* | *1* | *1.86 [1.71, 2.04]* | *3.71* | *7.8×10^-34^* | *1.83 [1.68, 1.98]* | *1.4* | *0.0002* |
| Height, standing | *174.0 [166, 180]* | *0.014* | *1* | *177.0 [169, 185]* | *0.06* | *1.7×10^-38^* | *175.0 [167, 182]* | *0.02* | *0.0007* |
| Righ forearm girth, flexed | *31.0 [28.0, 33.0]* | *-0.012* | *1* | *32.0 [30.0, 35.0]* | *-0.032* | *1* | *32.0 [30.0, 35.0]* | *0.11* | *0.0001* |
| Left shin girth | *37.0 [36.0, 38.0]* | *0.001* | *1* | *38.0 [36.0, 40.0]* | *0.001* | *1* | *38.0 [36.0, 39.0]* | *0.032* | *1* |
| Right thigh girth | *56.0 [53.0, 58.0]* | *0.01* | *1* | *59.0 [56.0, 62.0]* | *0.124* | *9.1×10^-17^* | *57.0 [54.0, 60.0]* | *0.022* | *1* |
| Right forearm girth, relaxed | *28.0 [26.0, 30.0]* | *-0.036* | *1* | *30.0 [27.0, 32.0]* | *-0.008* | *1* | *30.0 [27.0, 32.0]* | *0.113* | *0.001* |
| Left-hand grip | *38.0 [32.0, 46.7]* | *0.007* | *1* | *40.0 [33.0, 50.0]* | *-0.015* | *0.96* | *44.0 [35.0, 53.5]* | *0.057* | *2.7×10^-15^* |
| Weist girth | *71.0 [68.0, 74.0]* | *-0.021* | *1* | *75.0 [71.0, 82.0]* | *0.091* | *4.1×10^-13^* | *75.0 [69.0, 79.0]* | *-0.022* | *1* |
| Right shin girth | *37.0 [36.0, 38.0]* | *0.059* | *1* | *38.0 [36.0, 40.0]* | *0.097* | *0.0001* | *38.0 [36.0, 39.0]* | *0.031* | *1* |
| Neck girth | *36.0 [33.0, 39.0]* | *0.05* | *1* | *36.0 [34.0, 39.0]* | *-0.047* | *1* | *36.0 [34.0, 40.0]* | *-0.034* | *1* |
| Righ-hand grip strength | *39.0 [34.0, 48.0]* | *-0.001* | *1* | *42.0 [34.5, 52.0]* | *-0.001* | *1* | *46.0 [36.5, 55.0]* | *0.05* | *2.3×10^-13^* |
| Chest girth during exhalation | *88.0 [85.0, 93.0]* | *0.0004* | *1* | *91.0 [85.0, 97.0]* | *-0.002* | *1* | *91.0 [86.0, 97.0]* | *0.0002* | *1* |
| Left thigh girth | *56.0 [53.0, 58.0]* | *0.014* | *1* | *59.0 [56.0, 62.0]* | *0.139* | *1.7×10^-19^* | *57.0 [54.0, 60.0]* | *0.017* | *1* |
| Adipose tissue percentage | *11.5 [9.2, 14.8]* | *-0.026* | *1* | *14.5 [9.3, 20.0]* | *0.04* | *0.005* | *12.1 [8.1, 16.4]* | *-0.032* | *0.365* |
| Visceral fat percentage | *23.4 [6.1, 31.8]* | *-0.003* | *1* | *28.1 [5.0, 49.6]* | *0.006* | *0.746* | *17.5 [3.0, 39.6]* | *-0.006* | *1* |
| Muscle mass | *31.2 [26.6, 35.5]* | *-0.001* | *1* | *34.1 [28.6, 40.7]* | *0.07* | *6.9×10^-11^* | *34.2 [28.6, 39.9]* | *0.055* | *4.9×10^-5^* |
| Hip-to-waist ratio | *0.79 [0.77, 0.80]* | *-6.9* | *0.32* | *0.81 [0.78, 0.85]* | *2.68* | *1* | *0.80 [0.77, 0.84]* | *-2.17* | *1* |
| Fatty mass | *7.5 [5.6, 9.1]* | *-0.05* | *1* | *10.0 [6.8, 14.17]* | *0.063* | *3.0×10^-5^* | *8.20 [5.50, 11.03]* | *-0.04* | *0.144* |

*p-value obtained in regression analysis, with sports category as case. other participants as control, and sex, age, and BMI.

**Table S2**. Comparison of the frequencies of occurrence of variants significant for at least one sports category among the study participants and in open databases

| **Variant** | **Significance for sports category** | **Allele frequency in controls** | **Allele sfrequncy in cases** | **Allele frequency in PVFDB*** | **Allele frequency in gnomAD** | **Allele frequency in older adults** |
| --- | --- | --- | --- | --- | --- | --- |
| rs138125544 | Multi-sport | *0.011* | *0.059* | *0.011* | *0.008* | *0.011* |
| rs151237543 | Multi-sport | *0.011* | *0.064* | *0.011* | *0.008* | *0.011* |
| rs180831871 | Multi-sport | *0.011* | *0.064* | *0.011* | *0.008* | *0.011* |
| rs144380919 | Multi-sport | *0.009* | *0.054* | *0.009* | *0.020* | *0.009* |
| rs148699337 | Multi-sport | *0.014* | *0.064* | *0.014* | *0.019* | *0.013* |
| rs77475812 | Multi-sport | *0.01* | *0.059* | *0.008* | *0.018* | *0.008* |
| rs79605040 | Multi-sport | *0.009* | *0.059* | *0.009* | *0.018* | *0.008* |
| rs115472693 | Multi-sport | *0.01* | *0.059* | *0.009* | *0.018* | *0.008* |
| rs114773681 | Multi-sport | *0.015* | *0.069* | *0.014* | *0.026* | *0.015* |
| rs77411964 | Multi-sport | *0.01* | *0.056* | *0.009* | *0.018* | *0.009* |
| rs879783845 | Multi-sport | *0.008* | *0.049* | *0.009* | *0.067* | *0.008* |
| rs7226405 | Multi-sport | *0.048* | *0.147* | *0.051* | *0.000* | *0.054* |
| rs149865510 | Combat sports | *0.008* | *0.017* | *0.008* | *0.011* | *0.011* |
| rs1539441 | Combat sports | *0.074* | *0.115* | *0.078* | *0.106* | *0.073* |
| rs11599072 | Combat sports | *0.146* | *0.218* | *0.148* | *0.140* | *0.142* |
| rs12577360 | Combat sports | *0.007* | *0.023* | *0.007* | *0.013* | *0.009* |
| rs144318129 | Coordination-based sports | *0.008* | *0.025* | *0.009* | *0.017* | *0.010* |
| rs117223843 | Coordination-based sports | *0.01* | *0.031* | *0.011* | *0.007* | *0.013* |
| rs192088146 | Coordination-based sports | *0.01* | *0.03* | *0.011* | *0.007* | *0.013* |
| rs534226114 | Coordination-based sports | *0.009* | *0.027* | *0.010* | *0.012* | *0.013* |
| rs2058218638 | Coordination-based sports Game-oriented sports | *0.015* | *0.037* | *0.016* | *0.002* | *0.014* |
| rs1385732528 | Coordination-based sports | *0.012* | *0.033* | *0.016* | *0.500* | *0.011* |
| rs75413073 | Cycling sports | *0.011* | *0.03* | *0.012* | *0.003* | *0.022* |
| rs562552210 | Cycling sports | *0.007* | *0.021* | *0.008* | *0.002* | *0.008* |
| rs775815705 | Cycling sports | *0.126* | *0.941* | *0.062* | *0.264* | *0.126* |
| rs867704466 | Speed- and power-based sports | *0.013* | *0.055* | *0.011* | *0.000* | *0.010* |
| rs144534951 | Speed- and power-based sports | *0.009* | *0.045* | *0.009* | *0.014* | *0.009* |
|  | Speed- and power-based sports | *0.022* | *0.075* | *0.025* | *0.031* | *0.033* |
| rs149704888 | Speed- and power-based sports | *0.009* | *0.045* | *0.009* | *0.008* | *0.010* |
| rs1905446 | Speed- and power-based sports | *0.014* | *0.058* | *0.015* | *-* | *0.011* |
| rs189583925 | Speed- and power-based sports | *0.008* | *0.045* | *0.009* | *0.009* | *0.009* |
| rs185777639 | Speed- and power-based sports | *0.011* | *0.051* | *0.012* | *0.012* | *0.013* |
| rs71010624 | Game-oriented sports | *0.125* | *0.085* | *0.090* | *0.003* | *0.127* |
| rs1180116457 | Game-oriented sports | *0.009* | *0.022* | *-* | *-* | *0.011* |
| rs1029104728 | Game-oriented sports | *0.01* | *0.024* | *0.012* | *0.500* | *0.010* |

*PVFDB: database of the Federal Medical Biological Agency (FMBA of Russia) of variant frequencies in the Russian population [Application version 1.0.3 from December 5,.2024. Database version 59.1 from October 3, 2024; https://gdbpop.nir.cspfmba.ru/»]
