# Supplementary material for: GWAS-based polygenic profiling across broad sport categories: shared and category-specific genetic associations with athlete status and polygenic differences from population controls
Source: Front Sports Act Living. 2026 Sep 30;8:1813231. doi: 10.3389/fspor.2026.1813231 (PMC13663180; doi:10.3389/fspor.2026.1813231)
Supplement: Supplementary file 4 [file Datasheet4.docx]

**Table S3**. Predictors in the polygenic score model for multi-sports

| **Predictor** | **rsID or predictor description** | **Regression coefficient** |
| --- | --- | --- |
| pc1 | Principal component 1 | *-0.102700501395809* |
| GT_15_93389266_T_TA | rs10664640 | *0* |
| GT_2_65796386_A_C | rs76702946 | *0* |
| GT_9_10907333_G_C | rs142108776 | *0* |
| GT_5_65828877_T_C | rs78859670 | *0* |
| GT_19_32171217_A_AT | rs577868161 | *0* |
| GT_21_39314591_G_A | rs186970062 | *0* |
| GT_1_10261270_AT_A | rs141281398 | *0* |
| GT_21_39159259_A_G | rs117823642 | *0* |
| sex | Binarized sex (0 for women, 1 for men) | *0* |
| pc0 | Principal component 0 | *0* |
| pc2 | Principal component 2 | *0* |
| pc3 | Principal component 3 | *0* |
| pc4 | Principal component 4 | *0* |
| pc5 | Principal component 5 | *0* |
| pc6 | Principal component 6 | *0* |
| pc7 | Principal component 7 | *0* |
| pc8 | Principal component 8 | *0* |
| GT_6_158828652_CTTCT_C | rs536405163 | *0.075511603786428* |
| GT_1_208769503_A_G | rs79691749 | *0.08240199699622* |
| GT_1_208776178_G_A | rs1166937 | *0.08240199699622* |
| GT_8_126047028_G_A | rs192311062 | *0.088654847757778* |
| GT_7_18034636_G_A | rs79572696 | *0.098213127297971* |
| GT_4_68879536_T_C | rs117290547 | *0.102909352525092* |
| GT_4_68837229_T_G | rs116943623 | *0.102909352525092* |
| GT_4_68830168_T_G | rs141570585 | *0.102909352525092* |
| GT_4_68832236_G_C | rs140323822 | *0.102909352525092* |
| GT_4_68867310_C_A | rs118080525 | *0.102909352525092* |
| GT_4_68841792_A_AT | rs143357300 | *0.102909352525092* |
| GT_5_127569630_G_A | rs141588150 | *0.129834374959223* |
| GT_5_127569735_C_T | rs139706369 | *0.129834374959223* |
| GT_5_127570398_C_G | rs116243059 | *0.129834374959223* |
| GT_5_127571304_A_G | rs73783917 | *0.129834374959223* |
| GT_5_127570439_T_C | rs75240344 | *0.129834374959223* |
| GT_5_127571842_T_G | rs73783918 | *0.129834374959223* |
| GT_5_127571388_G_T | rs55654285 | *0.129834374959223* |
| GT_5_127573369_T_C | rs6894854 | *0.129834374959223* |
| GT_5_127576023_A_G | rs141448975 | *0.129834374959223* |
| GT_5_127570361_A_T | rs112585978 | *0.129834374959223* |
| GT_5_127572618_T_C | rs61220586 | *0.129834374959223* |
| GT_5_127569126_A_G | rs73783914 | *0.129834374959223* |
| GT_5_127568337_C_A | rs6875028 | *0.129834374959223* |
| GT_5_127569243_A_G | rs77483030 | *0.129834374959223* |
| GT_5_127567573_C_G | rs73783910 | *0.129834374959223* |
| GT_5_127567385_C_G | rs73783909 | *0.129834374959223* |
| GT_5_127567338_G_A | rs73783908 | *0.129834374959223* |
| GT_5_127567162_A_G | rs58380010 | *0.129834374959223* |
| GT_5_127567111_C_T | rs55749092 | *0.129834374959223* |
| GT_5_127574783_A_T | rs6879657 | *0.129834374959223* |
| GT_5_127567644_C_T | rs73783911 | *0.129834374959223* |
| GT_5_127568965_C_T | rs73783913 | *0.129834374959223* |
| GT_5_127568384_AAGGAGG_A | rs10562578 | *0.129834374959223* |
| GT_5_127568124_A_T | rs73783912 | *0.129834374959223* |
| GT_13_102438822_C_T | rs141181965 | *0.140721817585314* |
| GT_1_208786409_TAGG_T | rs67726419 | *0.147111717608684* |
| GT_6_158880535_G_A | rs9457492 | *0.153164363750213* |
| GT_6_158873306_T_C | rs9457486 | *0.153164363750213* |
| GT_8_126105234_C_G | rs573347138 | *0.153729890002434* |
| GT_8_126103312_A_G | rs150419855 | *0.153729890002434* |
| GT_22_50175957_G_A | rs182716744 | *0.174001969437758* |
| GT_6_158878534_T_A | rs13437571 | *0.192733908038379* |
| GT_3_122988759_C_T | rs137879511 | *0.211527506806179* |
| GT_4_68971770_T_C | rs117932473 | *0.215867058727006* |
| GT_22_50217114_G_A | rs34163881 | *0.219663293902751* |
| GT_22_50195007_C_T | rs145870754 | *0.219663293902751* |
| GT_1_105892845_A_G | rs878896457 | *0.245750596942567* |
| GT_13_106898712_GT_G | rs141722005 | *0.260634715585151* |
| pc9 | Principal component 9 | *0.261683549108115* |
| GT_5_127568815_G_GAA | rs10651113 | *0.264574463385765* |
| GT_22_37674552_T_C | rs113294619 | *0.277139663259878* |
| GT_22_37679827_G_A | rs41283239 | *0.277139663259878* |
| GT_22_37676678_C_G | rs79290987 | *0.277139663259878* |
| GT_10_25714274_A_T | rs144380919 | *0.281307382278761* |
| GT_5_94211375_C_T | rs78158324 | *0.293342969851351* |
| GT_5_94225392_A_C | rs191289436 | *0.293342969851351* |
| GT_12_66092983_T_G | rs117006610 | *0.29533155120382* |
| GT_14_27243147_G_A | rs1500439 | *0.297185994180956* |
| GT_3_117447614_C_T | rs79605040 | *0.30152982536579* |
| GT_3_117435496_C_T | rs77475812 | *0.30152982536579* |
| GT_3_117460503_A_G | rs115472693 | *0.30152982536579* |
| GT_3_117488016_C_T | rs77411964 | *0.30152982536579* |
| GT_14_95771237_G_A | rs79948978 | *0.311347199830381* |
| GT_2_137783901_G_T | rs141125266 | *0.320806534756608* |
| GT_5_170815047_C_A | rs190046334 | *0.320977412032074* |
| GT_3_117463165_A_C | rs114773681 | *0.321613920660791* |
| GT_8_126251128_A_G | rs549609234 | *0.323537371812785* |
| GT_16_24771571_G_GTT | rs879783845 | *0.324870462345631* |
| GT_4_179677720_A_G | rs138125544 | *0.335188740351631* |
| GT_2_137908571_C_T | rs184882426 | *0.344112807213961* |
| GT_2_137866136_C_G | rs149516613 | *0.344112807213961* |
| GT_16_83401659_C_CA | rs60316291 | *0.344647183889891* |
| GT_4_179699161_A_G | rs151237543 | *0.349083320167227* |
| GT_4_179711631_T_C | rs180831871 | *0.349083320167227* |
| GT_3_29786098_C_CA | rs368403371 | *0.356347590521682* |
| GT_4_68777200_G_GA | rs775428195 | *0.406813185529809* |
| GT_2_217762856_A_G | rs554636156 | *0.429539436589677* |
| GT_4_78633937_C_T | rs150895578 | *0.4607499723864* |
| GT_3_44008435_A_G | rs4588339 | *0.464835012065099* |
| GT_3_12593673_G_A | rs73019148 | *0.475564434657269* |
| GT_12_111426385_C_CT | rs796965805 | *0.482923175719224* |
| GT_6_158563136_C_T | rs148935794 | *0.518474565243196* |
| GT_4_5464924_T_A | rs114045090 | *0.523806717524148* |
| GT_20_21882971_C_T | rs75954625 | *0.525705902052437* |
| GT_6_167772682_CA_C | rs367581902 | *0.534016499793178* |
| GT_3_44009227_C_T | rs148699337 | *0.57235660824738* |
| GT_15_32061251_G_A | rs72713557 | *0.594648905821578* |
| GT_20_50765221_T_G | rs142368259 | *0.614456431981897* |
| GT_3_62206348_G_T | rs77308150 | *0.630761819315833* |
| GT_6_28680207_G_A | rs149912748 | *0.657824357402995* |
| GT_3_106821031_C_T | rs184305701 | *0.698637958440804* |
| GT_16_83414627_T_C | rs9940088 | *0.706354775362788* |
| GT_1_75600942_G_A | rs55834935 | *0.721719520526885* |
| GT_9_129542432_A_ATATATT | rs558014251 | *0.766576348111761* |
| GT_18_3438596_A_C | rs7226405 | *0.845046048363969* |
| intercept | | *-2.586387911948724* |

**For all SNPs included in the model, the predictor names are formatted as GT_[Chromosome]_[Position]_[Reference allele]_[Alternative allele] according to the GRCh38 reference genome. Pc0, pc1, pc2, etc, are the principal components with their corresponding numbers.*

**Table S4**. Predictors in the polygenic score model for combat sports.

| **Predictor** | **rsID or predictor description** | **Regression coefficient** |
| --- | --- | --- |
| GT_13_62912012_G_A | rs113265303 | *-1.06622021025164* |
| GT_4_40394680_T_C | rs11731787 | *-0.78367408429778* |
| GT_4_161714401_C_G | rs7693232 | *-0.659182279609671* |
| GT_1_62533688_A_G | rs78287364 | *-0.547199167228999* |
| GT_8_27335922_T_C | rs78711129 | *-0.516021919230135* |
| GT_12_91473661_G_C | rs75884356 | *-0.387679079390992* |
| GT_6_3739637_C_G | rs11242870 | *-0.319283183185393* |
| GT_13_62935754_C_A | rs73200898 | *-0.213153823203156* |
| GT_13_62952182_T_C | rs17251632 | *-0.213153823203156* |
| GT_13_62939483_A_G | rs73200900 | *-0.213153823203156* |
| GT_13_62945389_C_T | rs11841177 | *-0.213153823203156* |
| GT_13_62922893_T_C | rs34985690 | *-0.213153823203156* |
| GT_12_93516721_G_A | rs7306206 | *-0.197014447122619* |
| GT_13_62876387_C_G | rs35428785 | *-0.188234306423582* |
| pc2 | Principal component 2 | *-0.042946718916617* |
| pc5 | Principal component 5 | *0* |
| pc7 | Principal component 7 | *0* |
| GT_13_62892109_G_A | rs7988991 | *0.005659622247419* |
| GT_13_62894285_T_C | rs17087776 | *0.005659622247419* |
| GT_13_62896187_A_G | rs17087779 | *0.005659622247419* |
| GT_13_62897794_G_A | rs17087780 | *0.005659622247419* |
| GT_13_62893592_G_A | rs73188143 | *0.005659622247419* |
| GT_13_62891912_G_A | rs7988608 | *0.005659622247419* |
| GT_13_62901411_G_T | rs7331616 | *0.005659622247419* |
| GT_13_62895205_C_T | rs7985541 | *0.005659622247419* |
| pc3 | Principal component 3 | *0.006148398843274* |
| pc4 | Principal component 4 | *0.022650430842037* |
| pc0 | Principal component 0 | *0.026812374794874* |
| pc6 | Principal component 6 | *0.042357514594984* |
| GT_6_165008746_A_G | rs117019710 | *0.049188140738837* |
| GT_6_165009147_C_A | rs79861760 | *0.049188140738837* |
| pc8 | Principal component 8 | *0.077401529601505* |
| pc1 | Principal component 1 | *0.088500443816832* |
| pc9 | Principal component 9 | *0.090424450139248* |
| GT_1_117681976_T_C | rs1539441 | *0.198188617476015* |
| GT_6_165034401_C_T | rs79233099 | *0.244851473534169* |
| GT_12_91591305_T_C | rs57841816 | *0.258615547086997* |
| GT_10_5052195_G_A | rs11599072 | *0.358533173828929* |
| GT_3_64948751_G_GA | rs369827578 | *0.382655677922528* |
| GT_12_93511432_A_T | rs6538427 | *0.47380786855563* |
| GT_13_92298487_G_A | rs78980793 | *0.583330099121966* |
| GT_1_117644662_A_T | rs114352057 | *0.592088256126646* |
| GT_11_122382932_C_T | rs12365679 | *0.645519448743343* |
| GT_1_62481558_A_C | rs79547769 | *0.742850809042947* |
| GT_10_62757407_G_C | rs140719253 | *0.744105473606756* |
| sex | Binarized sex (0 for women, 1 for men) | *0.817774089541513* |
| GT_3_32417048_A_G | rs115963670 | *0.826693048866845* |
| GT_12_91554735_G_C | rs10859168 | *0.832232893392884* |
| GT_20_29862397_A_T | rs1169001546 | *0.900491929976899* |
| GT_11_85829059_T_G | rs12577360 | *1.00788446675928* |
| GT_7_157837239_A_G | rs111142055 | *1.00998886315547* |
| GT_4_40394831_A_G | rs11733856 | *1.01266105607728* |
| GT_4_161716902_A_G | rs72973247 | *1.22740706524723* |
| GT_8_27382649_G_A | rs78877121 | *1.35512728841813* |
| GT_5_125304962_G_T | rs149865510 | *1.43858271658017* |
| GT_13_62918312_G_A | rs7997350 | *1.69566402972121* |
| intercept | | *-1.3273371455615366* |

**For all SNPs included in the model, the predictor names are formatted as GT_[Chromosome]_[Position]_[Reference allele]_[Alternative allele] according to the GRCh38 reference genome. Pc0, pc1, pc2, etc, are the principal components with their corresponding numbers.*

**Table S5**. Predictors in the polygenic score model for coordination-based sports

| **Predictor** | **rsID or predictor description** | **Regression coefficient** |
| --- | --- | --- |
| GT_8_87094828_A_C | rs554334107 | *-4.99062266826301* |
| GT_6_170155195_T_C | rs71536938 | *-0.429578161153042* |
| GT_22_48679098_T_C | rs775815705 | *-0.423165475711978* |
| GT_6_170079158_C_CG | rs71010624 | *-0.204090865026699* |
| pc5 | Principal component 5 | *-0.150954257989827* |
| sex | Binarized sex (0 for women, 1 for men) | *-0.139729056702215* |
| pc1 | Principal component 1 | *-0.056782356656622* |
| pc0 | Principal component 0 | *-0.038031985798547* |
| pc8 | Principal component 8 | *-0.027478213760117* |
| pc2 | Principal component 2 | *-0.002183188082014* |
| pc3 | Principal component 3 | *0.00579395361503* |
| pc4 | Principal component 4 | *0.024180031980874* |
| pc6 | Principal component 6 | *0.040398630658817* |
| pc7 | Principal component 7 | *0.05032007243424* |
| GT_8_87228499_G_C | rs534226114 | *0.063925444140126* |
| pc9 | Principal component 9 | *0.09709886770312* |
| GT_10_90290358_G_A | rs80175269 | *0.279704141473556* |
| GT_10_90293857_T_C | rs77949634 | *0.279704141473562* |
| GT_10_90288091_G_A | rs78866702 | *0.279704141473565* |
| GT_15_43554620_T_TAC | rs757647925 | *0.356668287822428* |
| GT_8_87225612_C_G | rs192088146 | *0.35961660380131* |
| GT_8_87135486_A_G | rs117223843 | *0.359616603801315* |
| GT_11_13133286_T_C | rs61878011 | *0.407925627066661* |
| GT_11_13132966_A_G | rs61878010 | *0.407925627066667* |
| GT_6_31953291_T_G | rs1180007400 | *0.639184767812419* |
| GT_20_30934072_C_A | rs1385732528 | *0.720815971676779* |
| GT_19_2543221_CT_C | rs34642408 | *0.747898936883628* |
| GT_5_174214486_T_C | rs79152186 | *0.804248276191272* |
| GT_11_131851536_T_C | rs10894460 | *0.823510194069224* |
| GT_2_173166007_A_G | rs75365412 | *0.858180036812125* |
| GT_7_70725422_A_G | rs75254148 | *0.8719841343372* |
| GT_7_57445034_TAC_T | rs1423590307 | *0.893918044111961* |
| GT_4_169041979_C_T | rs144318129 | *1.02809253365557* |
| GT_13_113876201_T_A | rs373450272 | *1.08013240782282* |
| GT_18_7086629_A_C | rs2058218638 | *1.10293985986612* |
| GT_9_131507586_A_T | rs138335760 | *1.12722861604975* |
| GT_20_30827620_G_A | rs2124430 | *1.14044692747187* |
| GT_8_86992062_T_G | rs150629683 | *5.35558914396456* |
| intercept | | *-0.43532030886027173* |

**For all SNPs included in the model, the predictor names are formatted as GT_[Chromosome]_[Position]_[Reference allele]_[Alternative allele] according to the GRCh38 reference genome. Pc0, pc1, pc2, etc, are the principal components with their corresponding numbers.*

**Table S6**. Predictors in the polygenic score model for cycling sports.

| **Predictor** | **rsID or predictor description** | **Regression coefficient** |
| --- | --- | --- |
| GT_1_31997053_G_T | rs72666722 | *-0.90165976637091* |
| GT_11_69076977_A_G | rs111200393 | *-0.65880834477879* |
| GT_22_48679098_T_C | rs775815705 | *-0.355319507677224* |
| pc5 | Principal component 5 | *-0.15839870150327* |
| pc0 | Principal component 0 | *-0.115999522467473* |
| pc8 | Principal component 8 | *-0.111901025313872* |
| pc1 | Principal component 1 | *-0.08297563437915* |
| pc3 | Principal component 3 | *-0.013703004333936* |
| pc2 | Principal component 2 | *0.007514231748382* |
| pc9 | Principal component 9 | *0.02003518077352* |
| pc6 | Principal component 6 | *0.022130711568918* |
| pc4 | Principal component 4 | *0.026101089142024* |
| pc7 | Principal component 7 | *0.043815973320611* |
| GT_6_154227910_T_TA | rs549854199 | *0.483781592467672* |
| GT_6_154218025_A_G | rs189698711 | *0.483781592467672* |
| GT_22_21581800_C_CA | rs1226304090 | *0.559914618477072* |
| sex | Binarized sex (0 for women, 1 for men) | *0.563991754035236* |
| GT_1_199484668_A_AAT | rs144269945 | *0.594524749362422* |
| GT_2_164238446_G_C | rs562552210 | *0.639147694284787* |
| GT_16_36124946_G_T | rs1254047458 | *0.725672748549138* |
| GT_20_30934109_A_T | rs1456315862 | *0.867772076459904* |
| GT_4_10460338_A_ACC | Not found | *0.889270779263377* |
| GT_4_189280033_G_GC | rs66536845 | *0.977190914230588* |
| GT_16_17368602_A_AC | Not found | *1.17487037897168* |
| GT_5_152479947_C_G | rs75413073 | *1.34901915777165* |
| GT_13_92824726_G_A | rs1186519514 | *1.37814406793592* |
| GT_1_32182366_G_A | rs72666778 | *1.55122799512963* |
| intercept | | *-0.5622267715873832* |

**For all SNPs included in the model, the predictor names are formatted as GT_[Chromosome]_[Position]_[Reference allele]_[Alternative allele] according to the GRCh38 reference genome. Pc0, pc1, pc2, etc, are the principal components with their corresponding numbers.*

**Table S7**. Predictors in the polygenic score model for game-oriented sports

| **Predictor** | **rsID or predictor description** | **Regression coefficient** |
| --- | --- | --- |
| GT_11_69076977_A_G | rs111200393 | *-0.263216934945755* |
| GT_6_170079158_C_CG | rs71010624 | *-0.190226592409403* |
| pc5 | Principal component 5 | *-0.15541134828859* |
| GT_22_48679098_T_C | rs775815705 | *-0.154837944397235* |
| GT_1_238653021_G_A | rs61829326 | *-0.080207889485982* |
| GT_1_238658292_G_A | rs9662746 | *-0.076740116394429* |
| GT_1_238632594_A_T | rs56277513 | *-0.076316911473785* |
| pc1 | Principal component 1 | *-0.068042224078543* |
| GT_1_238632525_C_T | rs56183218 | *-0.059176687201905* |
| pc0 | Principal component 0 | *-0.057478462608372* |
| GT_1_238640886_T_G | rs61829325 | *-0.045842055501211* |
| GT_1_238654547_A_G | rs74699837 | *-0.03955858225728* |
| GT_1_238653862_C_T | rs10495418 | *-0.038244680889822* |
| GT_1_238617199_CAAAT_C | rs76814264 | *-0.034485173421265* |
| GT_1_238622400_C_T | rs61829321 | *-0.018487968726591* |
| GT_1_238623937_A_G | rs61829322 | *-0.012670865006564* |
| pc2 | Principal component 2 | *0* |
| pc3 | Principal component 3 | *0* |
| pc4 | Principal component 4 | *0* |
| pc6 | Principal component 6 | *0* |
| pc7 | Principal component 7 | *0* |
| pc8 | Principal component 8 | *0.019645576414306* |
| pc9 | Principal component 9 | *0.063946204556629* |
| GT_10_125896209_C_A | rs67429538 | *0.139812292314379* |
| GT_4_58246996_T_G | rs149992886 | *0.139821364928052* |
| GT_5_162364124_G_C | rs528735942 | *0.141016050869467* |
| GT_4_825979_G_A | rs191928157 | *0.146251668914133* |
| GT_4_58342769_G_T | rs17827220 | *0.149092694090603* |
| GT_10_43775360_G_A | rs558128236 | *0.169584236442686* |
| GT_10_43792672_C_T | rs575711159 | *0.171073294065561* |
| GT_13_71765591_G_A | rs73215247 | *0.181869824098416* |
| sex | Binarized sex (0 for women, 1 for men) | *0.20000089638665* |
| GT_8_55075004_C_CT | rs1180116457 | *0.206255869216128* |
| GT_20_30934072_C_A | rs1385732528 | *0.238712118937492* |
| GT_20_30933164_A_G | rs1039083409 | *0.244646881716336* |
| GT_1_29771466_A_C | rs111214081 | *0.267953064212571* |
| GT_20_30932028_A_T | rs1029104728 | *0.273307809035297* |
| GT_18_41514755_G_T | rs143584413 | *0.274708699906123* |
| GT_18_7086629_A_C | rs2058218638 | *0.282152126853749* |
| intercept | | *-0.15066152798359206* |

**For all SNPs included in the model, the predictor names are formatted as GT_[Chromosome]_[Position]_[Reference allele]_[Alternative allele] according to the GRCh38 reference genome. Pc0, pc1, pc2, etc, are the principal components with their corresponding numbers.*

**Table S8**. Predictors in the polygenic score model for speed- and power-based sports

| **Predictor** | **rsID or predictor description** | **Regression coefficient** |
| --- | --- | --- |
| GT_8_98993768_C_G | rs188907554 | *-0.389433944459911* |
| GT_8_99224758_G_C | rs117625198 | *-0.389433944459911* |
| GT_2_80419158_G_T | rs185777639 | *-0.263494108104574* |
| pc5 | Principal component 5 | *-0.217961741734017* |
| pc0 | Principal component 0 | *-0.01887125878765* |
| pc3 | Principal component 3 | *0* |
| pc4 | Principal component 4 | *0* |
| pc6 | Principal component 6 | *0* |
| pc7 | Principal component 7 | *0* |
| GT_5_60843233_G_A | rs138837208 | *0.048435012714063* |
| GT_5_60868998_T_G | rs114171222 | *0.048435012714063* |
| GT_5_61170432_A_G | rs115124790 | *0.048435012714063* |
| pc2 | Principal component 2 | *0.076041002484629* |
| pc1 | Principal component 1 | *0.078431904943888* |
| pc9 | Principal component 9 | *0.124096348572531* |
| GT_1_121448957_C_CA | rs34488438 | *0.14461911543801* |
| pc8 | Principal component 8 | *0.181963314157101* |
| GT_1_11007218_A_G | rs78022785 | *0.413417659716809* |
| sex | Binarized sex (0 for women, 1 for men) | *0.428944592234603* |
| GT_3_106035952_C_CA | rs145838624 | *0.45729980944357* |
| GT_12_67879719_T_G | rs1905446 | *0.457928161560035* |
| GT_19_46421765_C_A | rs144161850 | *0.49883279565025* |
| GT_11_90518893_C_G | rs867704466 | *0.518104735930749* |
| GT_1_120460112_T_G | rs1362549114 | *0.864074789807392* |
| GT_1_120434036_C_T | rs1553247417 | *0.864074789807392* |
| GT_1_120342703_G_T | rs1553236434 | *0.864074789807392* |
| GT_7_147495085_C_A | rs182619136 | *0.980526377477398* |
| GT_7_147344627_T_A | rs193181856 | *0.980526377477398* |
| GT_7_107944887_C_A | rs144534951 | *1.02470788221996* |
| GT_3_6347919_C_T | rs553296859 | *1.20857605875778* |
| GT_17_65574239_A_C | rs148557557 | *1.2137964946866* |
| GT_15_97996631_T_C | rs74706366 | *1.29612791547984* |
| GT_3_106126624_G_A | rs79277701 | *1.40985863899778* |
| GT_12_17815501_T_C | rs149205012 | *1.45686693951106* |
| GT_7_147128152_T_C | rs184090302 | *1.66960981891222* |
| GT_4_168733347_T_G | rs77206897 | *1.7917274081498* |
| GT_8_98771622_T_C | rs186668330 | *1.8990302225522* |
| GT_8_35312814_G_A | rs150672620 | *1.9190105487471* |
| GT_2_80374683_G_A | rs189583925 | *2.13775738428591* |
| GT_5_60789662_A_G | rs116618654 | *2.25567735634488* |
| GT_19_46452043_A_C | rs75783944 | *2.51674690346869* |
| GT_16_18978669_A_G | rs181980499 | *2.66181850388344* |
| GT_8_96154489_G_A | rs117797452 | *2.68805890658956* |
| GT_1_237594886_G_T | rs3121871 | *2.7043292034623* |
| GT_18_44978066_T_C | rs140262917 | *2.76859280876508* |
| GT_11_107700896_C_CA | rs58801703 | *2.83136818971723* |
| GT_14_100824324_C_T | rs191318372 | *3.30886948896353* |
| GT_7_146285316_T_C | rs752113472 | *3.99770000544039* |
| GT_6_39284373_G_A | rs149704888 | *4.89651263282976* |
| intercept | | *-3.2336477054681176* |

**For all SNPs included in the model, the predictor names are formatted as GT_[Chromosome]_[Position]_[Reference allele]_[Alternative allele] according to the GRCh38 reference genome. Pc0, pc1, pc2, etc, are the principal components with their corresponding numbers.*
